# The potential risks of nanomaterials: a review carried out for ECETOC

**DOI:** 10.1186/1743-8977-3-11

**Published:** 2006-08-14

**Authors:** Paul JA Borm, David Robbins, Stephan Haubold, Thomas Kuhlbusch, Heinz Fissan, Ken Donaldson, Roel Schins, Vicki Stone, Wolfgang Kreyling, Jurgen Lademann, Jean Krutmann, David Warheit, Eva Oberdorster

**Affiliations:** 1 Centre of Expertise in Life Sciences, Zuyd University, Heerlen, The Netherlands; 2 Cenamps, Newcastle upon Tyne, UK; 3 Nanogate Coating Systems, Saarbrücken, Germany; 4 IUTA, Duisburg, Germany; 5 ELEGI, University of Edinburgh, Edinburgh, Scotland, UK; 6 IUF at the University of Düsseldorf, Düsseldorf, Germany; 7 Dept of Biological Sciences, Napier University, Edinburgh, Scotland, UK; 8 GSF-Research Centre for Environment & Health, Institute of Inhalation Biology, Neuherberg, Germany; 9 Dermatology Clinic, Charite, Berlin, Germany; 10 Haskell Labs, Dupont de Nemours, Wilmington, USA; 11 Department of Biology, Southern Methodist University, Dallas, USA

## Abstract

During the last few years, research on toxicologically relevant properties of engineered nanoparticles has increased tremendously. A number of international research projects and additional activities are ongoing in the EU and the US, nourishing the expectation that more relevant technical and toxicological data will be published. Their widespread use allows for potential exposure to engineered nanoparticles during the whole lifecycle of a variety of products. When looking at possible exposure routes for manufactured Nanoparticles, inhalation, dermal and oral exposure are the most obvious, depending on the type of product in which Nanoparticles are used. This review shows that (1) Nanoparticles can deposit in the respiratory tract after *inhalation*. For a number of nanoparticles, oxidative stress-related inflammatory reactions have been observed. Tumour-related effects have only been observed in rats, and might be related to overload conditions. There are also a few reports that indicate uptake of nanoparticles in the brain via the olfactory epithelium. Nanoparticle translocation into the systemic circulation may occur after inhalation but conflicting evidence is present on the extent of translocation. These findings urge the need for additional studies to further elucidate these findings and to characterize the physiological impact. (2) There is currently little evidence from skin penetration studies that *dermal applications *of metal oxide nanoparticles used in sunscreens lead to systemic exposure. However, the question has been raised whether the usual testing with healthy, intact skin will be sufficient. (3) Uptake of nanoparticles in the gastrointestinal tract after *oral uptake *is a known phenomenon, of which use is intentionally made in the design of food and pharmacological components. Finally, this review indicates that only few specific nanoparticles have been investigated in a limited number of test systems and extrapolation of this data to other materials is not possible. Air pollution studies have generated indirect evidence for the role of combustion derived nanoparticles (CDNP) in driving adverse health effects in susceptible groups. Experimental studies with some bulk nanoparticles (carbon black, titanium dioxide, iron oxides) that have been used for decades suggest various adverse effects. However, engineered nanomaterials with new chemical and physical properties are being produced constantly and the toxicity of these is unknown. Therefore, despite the existing database on nanoparticles, no blanket statements about human toxicity can be given at this time. In addition, limited ecotoxicological data for nanomaterials precludes a systematic assessment of the impact of Nanoparticles on ecosystems.

## 1) Background

### 1.1 Definitions

Nanotechnology is considered by many as the next logical step in science, integrating engineering with biology, chemistry and physics [1]. It derives from the ongoing trend for miniaturisation in technology as described by Moore's Law and combination with other disciplines. Miniaturisation however has its limits and new approaches in manufacturing (bottom-up fabrication) have to be developed to reach anticipated milestones.

Nanotechnology can be considered as the application of science that "steps across the limit" of miniaturisation, where" new rules" become valid [2] More specifically, when the dimensions of a piece of solid material become very small, its physical and chemical properties can become very different from those of the same material in larger bulk form. This is one of the hallmarks of Nanotechnology, which can be described as a research area in which this limit of new properties is reached and strategies are developed to exploit the regime of size-controlled properties.

In the last couple of years, the term Nanotechnology has been inflated and has almost become synonymous for things that are innovative and highly promising. On the other hand it is also the subject of considerable debate regarding the open question on toxicological and environmental impact of Nanoparticles and nanotubes [3,4]. In this discussion a definition of Nanotechnology and its underlying sectors, applications and markets is important for the purpose of risk assessment and risk communication. Many definitions refer to the length scale (nano) of this new science but not all mention the new functionalities of materials and components at the nanoscale. A commonly-used working definition refers to the size and (changing) properties of materials in the size range between 1 nanometre (10 ) and 100 nm, but this gives rise to many uncertainties and inconsistencies which need to be resolved.

Although quite open and abstract the recent definition forwarded by a working group of the Europische Akademie [2] states:

"*Nanotechnology is dealing with functional systems based on the use of sub-units with specific size dependent properties of the individual sub-units or of a system of those"*

The report by the Royal Society and Royal Academy of Engineering [5] gives the following definitions of 'nanoscience' and 'nanotechnologies':

*"Nanoscience is the study of phenomena and manipulation of materials at atomic, molecular and macromolular scales, where the properties differ significantly from those at a larger scale"*;

*"Nanotechnologies are the design, characterisation, production and application of structures, devices and systems by controlling shape and size at nanometre scale"*.

Other definitions are more specific, such as the one used by Nanoforum:

Nanotechnology is made up of areas of technology where dimensions and tolerances in the range of 0.1 nm to 100 nm play a critical role

Or very simple, as defined by [6]*Nanotechnology – the manipulation, precision placement, measurement, modelling or manufacture of sub-100 nanometre scale matter*.

### 1.2 Major applications and markets in nanotechnology

The nanotechnology market can be broadly divided into 3 segments, viz. Materials, Tools and Devices:

**1. Nanomaterials **– used to describe materials with one or more components that have at least one dimension in the range of 1 to 100 nm and include Nanoparticles, nanofibres and nanotubes, composite materials and nano-structured surfaces. These include **Nanoparticles (NP) **as a subset of nanomaterials currently defined by consensus as single particles with a diameter < 100 nm. Agglomerates of NP can be larger than 100 nm in diameter but will be included in the discussion since they may break down on weak mechanical forces or in solvents. **Nanofibres **are a sub-class of nanoparticles (include nanotubes) which have two dimensions <100 nm but the third (axial) dimension can be much larger.

**2. Nanotools **– tools and techniques for synthesising nanomaterials, manipulating atoms and fabricating device structures, and – very importantly – for measuring and characterising materials and devices at the nanoscale;

**3. Nanodevices **– making devices at the nanoscale, important in microelectronics and optoelectronics at the present time, and at the interface with biotechnology where the aim is to mimic the action of biological systems such as cellular motors. This latter area is the most futuristic, and excites the greatest public reaction.

This report will primarily focus on the risks of **manufactured Nanoparticles and nanofibres**, because this area of nanotechnology should achieve volume production in the near term and it is also the aspect that raises greatest public concern about potential risks to health. However the distinction from composite nanomaterials and tools and devices becomes vague when nanoscale materials are combined for medical applications. In the nearer term, certain Nanoparticles offer opportunities to develop smart drug delivery vehicles that can move through the body to target sites, or sensor and diagnostic systems operating inside cells. Nanomaterials could also be used to synthesise structures for implant into the body that have properties that closely resemble the properties of natural materials. Tissue scaffolds that use biocompatible nanomaterials to control cell growth and adhesion are under development, and in the future artificial organs that mimic the porosity and capillary structure of natural organs such as the heart and liver may become reality [7]. These applications are not the subject of this study, but a working group of the European Science Foundation on this subject [7]. Nanomaterials constitute by far the most significant market opportunity in the foreseeable future. A 2002 market survey forecasts that by 2015 the total world industrial output in sectors likely to be influenced by nanomaterials will be in excess of $10,000 Billion (Realis ). The same survey suggests that by then about 10% of the output from the chemicals sector will be nano-influenced. The impact of nanotechnology will be seen particularly by enabling innovation in the areas of speciality and fine chemicals, and in materials for pharmaceuticals and personal care products. Nanoparticles and nanofibres will be particularly important in these applications.

The unique size-dependent properties of nanomaterials mean that in some ways they behave like new chemical substances. For example, Nanoparticles can scatter and absorb short-wavelength UV radiation but leave longer-wavelength visible light virtually unaffected. This property is exploited in transparent sunscreens. When fluorescent Nanoparticles absorb UV radiation they emit visible light, and the colour of the emitted light is different for Nanoparticles of different diameters. This effect is exploited when Nanoparticles are designed as colour-coded fluorescent labels that can be attached to target molecules or used as diagnostic markers. The changes in optical and transport properties become very pronounced for Nanoparticles smaller than about 30 nm. Particles in this range are often called 'quantum dots' because size is then controlling the separation (or quantisation) of energy levels inside the particle.

Some nanomaterials have been in volume production for a very long time. Carbon blacks in the nanoparticles size have been in production for more than a century, and are used for manufacture of rubber products and pigments. Fumed silica and other oxides such as titanium, alumina and zirconium have been produced as nanomaterials for over half a century and used as thixotropic agents in pigments and cosmetics, and more recently as the basis for fine polishing powders used in the microelectronics industry. Much of this high volume production is based on vapour phase flame or plasma reactions carried out under highly controlled conditions.

New nanomaterials are now being developed for many different applications, using new preparation techniques. Volumes are low, often on a laboratory scale producing <10 kg per day. Examples are magnetic materials for electric motors and generators as well as high density data storage, high current electrode materials for fuel cells, batteries and electrochromic displays, and materials with new surface properties for paints, coatings for self-cleaning windows, stain-resistant textiles etc. These materials are more expensive to produce than conventional materials, and must therefore offer very high performance or similar benefits to users to justify the additional cost. As manufacturing costs fall with increasing volumes and the maturing of nanotechnology methods, it can be predicted that the advantages of nanomaterials will lead to steady displacement of conventional materials in many high value applications.

**Surfaces **and **interfaces **are very important for new nanomaterials. As particles become smaller, the proportion of atoms found at the surface increases relative to the proportion inside its volume. This means that Nanoparticles can be more reactive, for example creating more effective catalysts or more efficient filler materials that allow weight reduction in composite materials. The higher surface energy can also make Nanoparticles interact strongly and stick together. If nanomaterial building blocks are synthesised in such a way that parts of the surface are sticky but other parts are passive and non-sticky, then random Brownian motion in a fluid can cause the blocks to stick together in defined ways to make larger structures. This is the basis of so-called 'bottom-up' self-assembly methods.

The chemicals industry is a key actor in exploiting nanotechnology because the nanomaterials that it produces are the basis for product innovation across many industrial sectors. The In Realis market survey highlights transportation equipment, electronic and electrical equipment, industrial machinery and instrumentation, rubber and plastic products, metal industries, and printing and publishing as sectors where nanotechnology is expected to have a large impact.

The study of materials such as ceramics, metals, colloids and polymers has always involved science at the nanoscale, and it is in these areas that the introduction of nanotechnology as an engineering discipline will have the earliest commercial impact. Advanced ceramics combine well-controlled microstructures with complex compositions and crystal structures. The use of nanoscale ceramic powders of narrow size distribution and high purity will allow compacting into ordered and uniform arrays, and sintering at lower temperatures, to produce tightly-controlled ceramic products for microelectronics, magnetic recording, chemical applications etc.

Nanocomposites combine polymers with nanomaterials to produce new thermoplastic and thermosetting materials. The high surface area-to-weight ratio for nanomaterials, the high surface reactivity and the matching of scale with polymer molecule dimensions can all contributes towards improving composite properties. For example, nanocomposites can have higher tensile strength or heat distortion temperature than similar materials using conventional fillers. These properties might be achieved with lower loading of nanomaterial filler, reducing the weight and increasing the transparency of nanocomposites compared with conventional composites. The use of carbon nanomaterials such as C_60 _fullerenes and nanotubes is a particularly promising way of reinforcing composite materials and perhaps making them lighter, stronger and electrically and optically active at the same time.

### 1.3 Standards and terminology

It is widely accepted that there is an urgent need for standards in nanotechnology to support legislation and regulation, risk analysis and communication, IP protection, and methods for sampling and measurement. In October 2004 the Technical Board Working Group on Nanotechnologies of the European Committee for Standardisation (CEN/BT/WG 166) launched a stakeholder consultation involving questionnaires for industry and non-industry groups.

The American National Standards Institute  has formed a Nanotechnology Standards Panel (ANSI-NSP), which issued a set of priority recommendations. Four areas were deemed to be most urgent for the next 12 months, and within each area 3 topics were identified as having greatest importance. These groupings of priorities for nanotechnology standardisation are:

Group 1: Systematic terminology for materials composition and features

• Composition

• Morphology

• Size

Group 2: General terminology for nanoscience and technology

• Definition of the term 'nano'

• Consideration of impact on intellectual property/other issues

• Sensitivity to existing conventions

Group 3: Metrology/methods of analysis/standard test methods

• Particle size and shape

• Particle number and distribution

• Particle mass

Group 4: Toxicity effects/environmental impact/risk assessment

• Environmental health and safety

• Reference standards for testing and controls

• Testing methods for toxicity

Standards for the areas of 'Manufacturing and Processing' and for 'Modelling and Simulation' were considered less urgent (3–5 year timeframe).

As outlined in Group 4 above, the greatest current risk is to the occupational health of workers involved in research and manufacture of Nanoparticles and nanofibres. However, as applications of nanomaterials increase, the risk of exposure to the general public will grow. It will be necessary to monitor products that incorporate Nanoparticles and nanofibres throughout their life, from manufacture to disposal, in order to estimate the probability of environmental emissions particularly from disposal and waste management processes. Some products will involve direct delivery of Nanoparticles to humans, for example injection of smart drug delivery systems and diagnostic markers and application of cosmetics to the skin. In some cases there could be unintentional uptake, for example ingestion of Nanoparticles used in food packaging technology.

The wide variety of routes by which Nanoparticles could be taken up by the body complicates the definition of Nanoparticles to be used in risk assessment and regulation. It is probably necessary to consider multi-component and multi-phase particles of any size and composition that can be absorbed by the body and then break up to deliver Nanoparticles or nanofibres to target organs. Nanocoatings on implanted medical devices that could shed Nanoparticles or nanofibres through use or wear should also be included. The overall risk of exposure to nanofibres may be lower than for Nanoparticles because it appears to be more difficult to generate aerosols of nanofibres.

### 1.4 Sources of nanoparticles

#### 1.4.1. Unintentionally produced nanoparticles

Epidemiological studies consistently show that increases in atmospheric particulate concentrations lead to short-term increases in morbidity and mortality. Inhalation is the most significant exposure route for those unintentionally- generated particles,. Regulation is aimed mainly at particulate matter <10 μm in diameter (PM_10_) in the atmosphere, but there is evidence that the adverse health effects are greater for the fine particle fraction, defined as having diameter <2.5 μm (PM_2.5_). Improvements in measurement techniques have focused attention more recently on ultrafine particles (UFPs), whose diameters < 0.1 μm (PM_0.1_) is consistent with nanoparticles definitions. UFPs dominate the number concentration of the ambient particle cloud, but represent only a small fraction of the total mass concentration. The large number concentration of UFPs existing in the atmosphere generates a background against which emissions of manufactured Nanoparticles and nanofibres will have to be measured and monitored. The relative importance of different sources of atmospheric UFP emission in the UK during 1996 was identified in the report of the Airborne Particles Expert Group [8]. The major source of primary UFP was road transport (60%), followed by combustion processes (23%, combining industrial, commercial, residential combustion and energy production).

Particles in the atmosphere are defined as either primary or secondary particles.

• Primary particles are emitted directly from sources or processes, which might be natural (fires, volcanoes, sea spray, erosion) or anthropogenic (traffic, industry).

• Secondary particles are formed in the atmosphere by gas-to-particle conversions. Immediately following nucleation the secondary particles are very small (~1–10 nm), and grow by coagulation or condense onto existing submicrometer particles. Homogenous nucleation, the formation of very small particles may occur in hot combustion gases and in metallurgical processes, including welding.

#### 1.4.2 Intentional production of nanoparticles

On the other hand, for decades industry has been intentionally producing different kinds of manufactured Nanoparticles, for use in existing applications such as pigments, resins and cosmetics. In addition, nanotechnology will increasingly generate new materials and products that are based on Nanoparticles, devices and tools. It is the sum of existing and newly developed, intentionally produced (= manufactured) Nanoparticles that form the primary target of risk assessment in this review.

It may be that the emission of manufactured Nanoparticles and nanofibres will add to the load of primary UFPs in the atmosphere. The subsequent formation of agglomerates of Nanoparticles and fibres, or of multi-component particulate matter containing adsorbed Nanoparticles, will add to the load of atmospheric PM_2.5 _or PM_10_. The formation of large complex particles incorporating nanoscale components will need to be considered in the risk assessment for nanotechnology because they create a potential route for uptake of Nanoparticles and fibres. The probability that emissions of manufactured Nanoparticles might occur where there are already high concentrations of UFPs in the atmosphere will require study to assess the rates of interaction between the different types of particle, and the likely products. Of course, the application of new manufactured Nanoparticles and nanofibres in products that are used directly by consumers means that other routes of uptake for manufactured nanomaterials must be considered in addition to inhalation, e.g. ingestion and dermal absorption.

**Figure 1:**
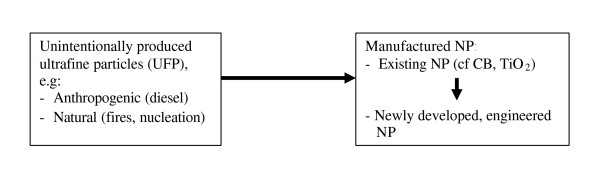
Illustration of the different sources and applications of ultrafine and Nanoparticles (NP). Epidemiology and toxicology have demonstrated acute effects of anthropogenic NP (= UFP) in humans, as well chronic effects of existing, manufactured NP in animals. It remains an open issue whether the hazards and risks found with those types of NP can be extrapolated to newly developed engineered NP.

For most manufactured NP no toxicity data are available. Most of the experimental, toxicological work on NP has been generated with a small set of Nanoparticles, such as carbon black (CB), titanium dioxide (TiO_2_), iron oxides and amorphous silica. These NP have been manufactured by the chemicals industry for some decades and are produced in many tons per year. These NP were considered to be so-called nuisance dusts until it was observed that upon prolonged exposure in rats, inflammation and lung tumours can occur. The discussion on the risks of manufactured NP is mainly driven by epidemiological studies that estimated that per 10 μg/m^3 ^increase in the concentration of environmental particles (PM_2.5_), overall mortality increases by 0.9%, while deaths from specific respiratory diseases can increase by as much as 2.7% [9]. Experimental toxicological studies (with CB, TiO_2_) have indicated that NP cause such adverse effects at lower dose levels than their fine counterparts, but so far few human studies have been able to investigate this. In summary, these findings set the stage for the current discussion on risks of Nanoparticles illustrated as the scheme in Figure 1. The key-question is whether and how the different pieces of toxicological and epidemiological evidence on different NP can be mutually used or whether a more targeted and systematic approach is necessary.

### 1.5 Production of manufactured nanoparticles

According to the National Nanotechnology Initiative (USA), the largest production volume in 2004 was for chemical-mechanical polisher (CMP) for semiconductor wafers, for example ammonia-stabilized 40 nm colloidal silica (sold under the name OX-200; no specific quantities given). Thousands of tons of silica, alumina and ceria, in the form of ultrafine abrasive particle mixtures that include Nanoparticles, are used each year in slurries for precision polishing of silicon wafers. Cabot Microelectronics, the leading producer of CMP slurries, recently announced that it has been working with Nano Products Corporation for 2 years to develop specially engineered Nanoparticles to improve the performance of next generation polishing products.

The manufacture of Fullerenes could soon match the engineered metal oxide Nanoparticles in production quantities, with the Kitakyushu plant (Mitsubishi, Japan) estimating an annual production of 1500 tonnes of C_60 _by 2007. Other manufacturing facilities also anticipate increased production of fullerenes, and therefore the sum production could be several thousands of tons of fullerenes by 2007. In 2003, Single-Walled and Multi-Walled Nanotubes had a world-wide production of approximately 3000 kg (3 tonnes). However, the Carbon Nanotechnology Research Institute (Japan) plans on expanding their production from ~ 1000 kg in 2003 to 120,000 kg per year within the next five years. The worldwide production *capacity *for single-wall and multi-wall carbon nanotubes is estimated to be about 100 tons in 2004 [10], increasing to about 500 tons in 2008. Capacity for single-wall nanotubes (SWNT) currently comprises less than 10% of the total, but is predicted to double as a proportion of total capacity by 2008.

The likely global production for specially-engineered nanomaterials is summarised in Table 1, using recently-published data [10] based on review of chemistry journals and market research:

**Table 1:** Estimated global production for engineered nanomaterials[11]

**Application**	**Nanomaterial/device**	**Estimated global production (tonnes per year)**		
		
		2003/04	2010	2020
Structural applications	Ceramics, catalysts, films & coatings, composites, metals	10	10^3^	10^4 ^– 10^5^
Skincare products	Metal oxides (eg. TiO_2_, ZnO)	10^3^	10^3^	10^3^
Information & Communication Technologies	SWNT, nanoelectronic and optoelectronic materials (excluding CMP slurries), organic light emitters and electronics, nanophosphors	10	10^2^	>10^3^
Biotechnology	Nanocomposites & encapsulates, targeted drug delivery, diagnostic markers, biosensors	<1	1	10
Environmental	Nanofiltration, membranes	10	10^2^	10^3 ^– 10^4^

The increase in production of engineered nanomaterials is being fuelled by a worldwide growth in R&D, which is seen as key to industrial innovation in many sectors. The scale of investment in nano-related R&D is illustrated by data taken from two recent publications, referenced in Tables 1.2 and 1.3.

Although current production of engineered nanomaterials is small, it is evident that as a result of increased R&D the rate of production will accelerate in the next few years. Considering the tons of engineered nanomaterial planned for production, it is likely that some of these materials will enter the environment during the product's Life Cycle (manufacture, use, disposal). In addition to these specifically engineered nanomaterials, it is estimated that 50,000 kg/year of nano-sized materials are being produced through these un-intended anthropogenic sources such as diesel-exhaust and other combustion processes. Given the large surface area to mass ratio of nano-sized materials, this is quite a large amount of reactive surface area.

### 1.6 Roadmaps for future manufacture and applications of nanomaterials

There are two distinct approaches to making products with nanoscale features and attributes.

**Top-down fabrication **is the method used in the microelectronics industry, where small features are created on large substrates by repeated pattern transfer steps involving lithographic methods. Extreme UV photolithography can produce patterns with feature sizes down to 100 nm, and electron beam lithography can be used for features down to 30 nm.

**Bottom-up fabrication **is directly relevant to the chemicals industry. This method starts with very small units, often individual molecules or even atoms, and assembles these building-block units into larger structures – clearly the domain of chemistry. What nanotechnology brings is the idea that the assembly can be hierarchical and controlled in specific ways. Some recent reports give a vision of how manufacture and applications of nanomaterials could evolve in the next 10–20 years:

'*Nanomaterials by Design' *[12]

This report was prepared by a working group representing the US chemical industry. It proposes actions that by 2020 will enable the industry to offer a library of nanomaterial building blocks with well-characterised compositions, stable architectures and predicted properties. There will be safe, reproducible and cost-effective 'bottom-up' manufacturing and assembly methods to incorporate these nanomaterial building blocks into devices and systems designed to perform specific functions whilst retaining the nanoscale attributes.

*'Vision 2020 – nanoelectronics at the centre of change' *[13] This report was prepared by representatives of European industrial and research organisations as a roadmap for development of microelectronics in Europe. Break-through applications enabled by nanomaterials could include new data storage devices, flexible displays, molecular transistors and novel sensor and actuator devices.

'*Nanotechnology – innovation for tomorrow's world' *[14]

The German Association of Engineers- Technology Centre (VDI-TZ) for the German government prepared this report. It describes the scientific background to nanotechnology developments and future areas of application. It contains many illustrations of how everyday objects and activities will be affected by the use of nanomaterials, and how this could change people's lives for the better.

## 2) Physico-chemical and surface properties of nanoparticles: aggregation and disaggregation

### 2.1 Properties of nanoparticles

Nanoparticles are unique since between 1 and 100 nm the physical behaviour of particles changes from classical physics to quantum physics with decreasing particle size. A nanoparticle with a radius of 2.5 nm and a density of 5 g/cm^3 ^has a surface of 240 m^2^/g when assuming a ball like shape. That means that around 20% of the particle atoms are at its surface. However, the surface of a nanoparticle is never "naked". Due to high energetic adhesive forces close to the surface, the particles are either agglomerated to their neighbours, glued to the next available surface or work like an activated charcoal filter towards other small molecules. Varying its composition, size or surface composition can therefore change the physical and chemical properties of a nanoparticle:

• **Size effects**: Depending on the material used to produce Nanoparticles, properties like solubility, transparency, colour, absorption or emission wavelength, conductivity, melting point and catalytic behaviour are changed only by varying the particle size.

• **Composition effects**: it is clear that different particle compositions lead to a different physical and chemical behaviour of the material.

• **Surface effects**: Properties like dispersibility, conductivity, catalytic behaviour and optical properties alter with different surface properties of the particle.

When it comes to the technical application of synthetic Nanoparticles, all those parameters need to be controlled. However, if the surface properties cannot be controlled, Nanoparticles quickly turn into larger particles due to agglomeration. Most of the size dependent effects are then lost. On the other hand biological adverse effects seem to be driven by the same exceptional effects of NP, that is large and active surface area [15,16]. The discussion whether single particles or agglomerates are important in these effects has not been resolved yet, but nanotubes ingested by macrophages were reported to be present as single particles based on their chemical properties [17].

**Figure 2:**
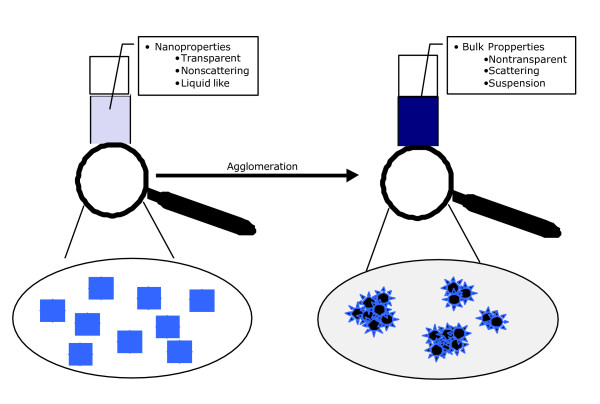
Dispersed Nanoparticles are needed in order to retain their specific properties for the technological applications.

This chapter will mainly deal with single and agglomerated particles from the point of view of synthesis and application. When it comes to the application of new properties of nanomaterials, synthesising Nanoparticles is not enough. It is their dispersion in the product that matters and makes Nanoparticles so attractive. When agglomerated, Nanoparticles loose those properties that are determined by size like colour and transparency. Since agglomerates tend to settle out they are difficult to be dispersed within a polymer matrix, an ink, paint, cream etc. For the application of Nanoparticles, it is therefore crucial to control their agglomeration behaviour. Dispersed Nanoparticles are needed in order to retain their specific properties for the technological applications (fig 2).

### 2.2 Synthesis of nanoparticles and their aggregation

From an application point of view dispersed Nanoparticles are ideal. Whether or not Nanoparticles can be dispersed or not strongly depends on the synthetic route chosen to produce it.

- **Milling**: Milling materials down to the nanometre regime belongs to the so-called top-down approach. Macro material is filled into a ball mill or something similar and can be milled down as far as around 30 nm. The advantage is the low cost of the procedure. One disadvantage is, that the grain boundaries are still touching and stick strongly together. Thus these particles are very difficult to disperse. Additionally high mechanical forces applied to the material can lead to a disruption of the lattice of the particles and which could dramatically change its properties.

- **Gas-Phase**: Gas-Phase procedures belong to the so-called bottom-up approach. Precursors of the end product are being reacted in the gas phase in a vacuum chamber and crystal growth is controlled by parameters like precursors, temperature and concentration. Anticipated advantages include high quality particles, mass produced fairly cheaply and with a highly defined particle size distribution. The main disadvantage is difficulty in controlling the surface of the Nanoparticles.

- **Wet-Phase**: The wet-phase procedure also belongs to the bottom-up approach and works only in solution. Molecules in the reaction mixture, temperature and reaction time control the crystal growth. The advantage of this procedure is the total surface control during every stage of the particle growth. Disadvantage is mostly price and scalability of the process. Mostly liquid phase synthesis leads to a dispersion of Nanoparticles. Since the solvent is either hydrophilic or hydrophobic, the application of such dispersion is limited. Latest developments however show that Nanoparticles agglomerated on purpose can be flocculated out of the liquid phase or stay perfectly dispersible in a solvent. Starting from a dispersible powder the surface composition can be chemically changed and adjusted to many different solvents and applications.

### 2.3 Synthesis of nanoparticles and their aggregation

Generally, most suppliers apply a post- synthetic strategy to modify Nanoparticles to prevent aggregation or stimulate disaggregation. Therefore particle powders are mechanically milled under the addition of dispersing additives. The additives form a layer around the particle and inhibit aggregation. If the synthetic route allows the introduction of surface molecules before agglomeration takes place, particles can be dispersible in certain media straight away. Additionally surface molecules can be changed by chemical synthesis after the particle has formed. This post synthetic route opens a variety of possible surface modifications, which can be adjusted to any application. In some cases especially in water, particles can be stabilized by their surface charge. This behaviour is influenced by the ion content of the water and the amount of charged ions on the surface of the particle.

There is a body of evidence from drug delivery and toxicological literature that surface modification as well as surface charge can have major impact on biological response to particles, including phagocytosis, genotoxicity, and inflammation. Coating of particles with polyethylene glycol is a common treatment in drug delivery to prevent recognition by the reticulo-endothelial system (Chapter 4) and increase the half-life of the particle conjugated drugs. Surface modified TiO_2 _has been the subject of considerable investigation which has shown that the hydrophobic coatings usually tend to lower the inflammatory response after inhalation or instillation [18,19]. Therefore, it is recommended that the surface modification and the agents used are vital information in the description of any nanoparticle.

### 2.4 How to assess the surface properties of nanoparticles

As discussed above the technical application of Nanoparticles mainly depend on their surface. It is therefore crucial to the chemist to control the surface and thus the properties of single particles. However, the qualitative and quantitative analysis of the surface of a single nanoparticle or a nanoparticle ensemble is challenging. The following methods are commonly used.

#### 2.4.1 Zeta potential

The zeta potential is a function of the surface charge of the particle or any adsorbed layer at the interface and the nature and composition of the surrounding medium in which the particle is suspended. It is usually of the same sign as the potential at the particle surface. The zeta potential is readily measurable by experiment. It reflects the effective charge on the particles and is therefore related to the electrostatic repulsion. The zeta potential is a relevant tool for the practical study and control of nanoparticulate dispersions. The Zeta potential however, gives no information on the chemical composition, or the elemental composition of the surface.

#### 2.4.2 Secondary ion mass spectroscopy (SIMS)

SIMS is a destructive method that gains information about the atomic compositions of layers from 1–3 nm with a high lateral resolution. SIMS and other similar methods however, can only give information about the elemental composition of a material and say nothing about the chemical properties like reactivity and binding states of the elements near the surface.

#### 2.4.3 X-ray photoelectron spectroscopy (ESCA)

Electron spectroscopy for chemical analysis (ESCA) is a non-destructive method used to measure the atomic composition of layers between 1–10 nm with a rather poor lateral resolution. It therefore is an excellent method to characterize the chemical composition of nanomaterials. ESCA gives information not just about the elemental composition of a material but also about the binding state of different elements. It is a sensitive method to measure the distribution of different elements down to 1 atom%.

#### 2.4.4 Thermogravimetry

In thermogravimetry, surface molecules are removed from a nanomaterial by slowly heating the material and measuring the change in weight. In addition mass spectroscopy can be used online to measure the type of molecule removed from the surface. In combination, both methods give a good picture of the molecules bound to the surface of a nanoparticle.

#### 2.4.5 Atomic force microscopy (AFM) and scanning tunnelling microscopy (STM)

AFM and STM are based on the use of a fine needle scanning over a given surface. This can be controlled even below atomic resolution. Thus they are powerful methods not only to measure structures and topographies of nanomaterials but also, in combination with chemical force microscopy, to identify single molecules on surfaces. The information about a single particle its chemistry, charge, magnetic properties etc. can be very high, provided that the particle can be fixed. The information about an ensemble of particles however, is poor.

#### 2.4.6. Particle surface reactivity

Rapid approaches that have been used to determine particle surface reactivity fro toxicology studies include e.g. testing of plasmid DNA unwinding or oxidation of calf thymus DNA [20,21]. Electron paramagnetic resonance (EPR) combined with a spin-trap has been used to determine the radical generation properties of particulate materials well above the nanosize range such as quartz and asbestos in relation to (surface) modification as well as of ambient particulate matter. In general, these EPR studies showed positive associations with toxicity in vitro and/or in vivo toxicity [22-24]. Nanoparticles such as fullerenes are well known to produce such reactive oxygen species in suspension [25,26] and these properties are considered relevant for both technological application and toxicological hazard.

## 3) Exposure potential during ongoing production and application: role of processes

### 3.1 Introduction

This chapter is related to the health and environmental safety of Nanomaterials. Therefore any discussion related to "combustion" Nanoparticles, such as diesel soot are not included. More specifically, the discussion is focussed on manufactured nanoparticles (NP) and their agglomerates being produced for direct or indirect commercial and/or industrial use. Agglomerates of NP can be larger than 100 nm in diameter but have to be included in the discussion since they may break down on weak mechanical forces or in solvents. No differentiation between agglomerates and aggregates is made here since no clear definition to distinguish those two exists.

The differentiation coming from engineering science is important for the risk assessment of NPs. Studies related to and a definition based on the stability of agglomerates during uptake by humans is important since it changes the active number and surface concentration and hence influences the dose response. The US EPA poses six primary questions that the exposure assessment should answer:

1. How does exposure occur?

2. What or who is exposed?

3. How much exposure occurs? When and where does it occur?

4. How does exposure vary?

5. How uncertain are exposure estimates?

6. What is the likelihood that exposure will occur?

A general systematic approach is introduced in paragraph 3.2, while paragraph 3.3 deals with the questions 1, 3, 6. Paragraph 3.4 gives a summary of the available instrumentations and method recommendation for NP exposure measurements, while 3.5 discusses questions 2, 4 and 5.

### 3.2 Process/product lines of nanoparticles and possible exposure

**Figure 3:**
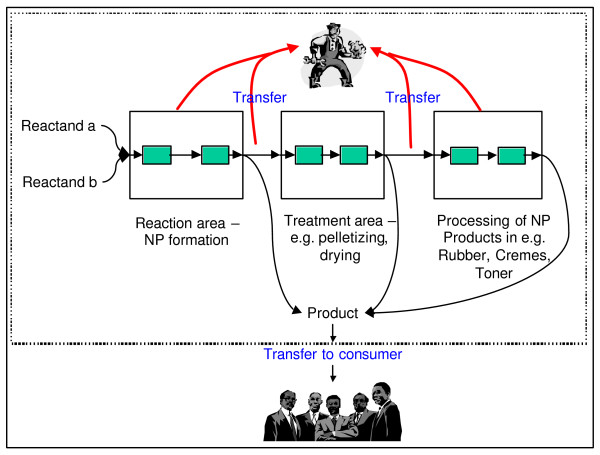
Scheme of NP production and possible exposure.

Figure 3 shows a general scheme on the production of NP. The boxes indicate different steps in the production, treatment and processing of NP. Within the boxes small, green boxes are included to denote closed cycles with no intentional release of product/by-product NP. Generally, release of NP can occur in closed cycles through e.g. leaks and during the transfer of the intermediate or final product to other handling steps. This exposure to NP is mainly related to workers since all these steps take place in working environments as indicated by the dashed line. Exposure of the public to NP can only occur through the product or release of NP's to the environment through stack or diffusive emissions.

Conclusions and recommendations:

- No systematic approach related to the control of NP-production and -products exists to our knowledge.

- Therefore the above or a similar scheme should be implemented for exposure and hence risk assessments for NP processes, treatment and products.

- Other recommendations related hereto are the expert recommendations 1 and 2 in Nanotechnologies [27], including Development of a nomenclature for intermediate and finished engineered nanomaterials as an international effort (expert recommendation 1) and Assignment of a universally recognized Chemical Abstract Service (CAS) Number to engineered NPs (expert recommendation 2).

- Sources of NPs not directly related to Nanomaterials or its production should be separated from the discussion as indicated in the expert recommendations 1 and 2 above. Still, they have to and will be included in the discussion of health effects induced by Nanoparticles.

### 3.3 Exposure potential to NP

Separate factors have to be investigated and discussed to assess the exposure potential. Rephrasing the questions given in the introduction the following relevant factors are identified:

- Probability of exposure

- Extent of exposure (time and concentration)

- Uptake route (inhalation, trans-dermal, ingestion)

No systematic approach is currently available assessing the probability of exposure related to NP production and handling processes. A systematic assessment of this could be based on the scheme presented in paragraph 3.2. Differentiation can be made e.g. between closed production cycles and "open" handling of the products/by-products. A systematic review on applied production methods, products and their handling must be compiled before any specific recommendations can be given related to the probability of exposure. Post-synthetic processing to achieve particle dispersion and surface-modifications (as described in Chapter 2) also need to be included in such a review.

Uptake of Nanoparticles by humans may occur by inhalation, trans-dermal or by ingestion. The currently mostly discussed and investigated exposure route is via inhalation. This route has to be treated differently to trans-dermal processes and ingestion. It is difficult tp assess the **personal exposure **to NP and ultrafine particles. No personal sampler exists to specifically measure the concentration (either mass or number concentration) of particles below 100 nm diameter. Current standard occupational exposure measurements are based on mass concentrations of the inhalable or alveolar particle size fraction. First measurements of number size distributions of particles at working places to assess the potential exposure were conducted at a few selected working places/areas. The exposure can currently be only deduced/calculated based on the limited measurements conducted so far.

Recommendations are:

- Develop a systematic review of applied and planned NP production methods, products and their handling.

- Develop a systematic approach in assessing the release potential related to NPs and their agglomerates.

- Develop a method to reproducibly assess personal exposure to NP and their agglomerates.

- Compile current data on number size distribution measurements in working areas and estimate personal exposure levels to derive an overview on possible exposure levels.

- Measure/Calculate exposure of the skin to Nanomaterials and especially NP for workers handling powder like Nanomaterials such as Carbon Black or Titanium dioxide.

- Development and promotion of good practices in handling Nanomaterials (see also expert recommendation 6 in BIA report on "ultrafine particles at working places .

### 3.4 Methods for characterization and assessments of exposure

#### 3.4.1 Which metric to use for NP exposure?

One of the key questions related to Nanomaterial exposure is the particle parameter to be measured. Possible parameters could be number concentration, surface area, mass concentrations, weighted size distribution, state of agglomeration, surface reactivity (e.g. ability to produce radicals, zeta potential), chemical composition, and morphology.

Due to the vast number of characteristics, which can be determined for Nanoparticles only a brief overview of measurement methods can be given. A technical report on "Occupational Ultrafine Aerosol Exposure Characterization and Assessment  is currently drafted and includes an extended review on particle characterization methods. It has to be noted that mass concentrations of ultrafine particles are generally extremely low and hence cannot easily be determined in current standard exposure measurements. Number concentrations, in contrast, are mainly dominated by particles in the ultrafine size range. Since the latter can be determined relatively easily by e.g. condensation nucleus counters. This is the main parameter currently used in exposure measurements.

**Figure 4:**
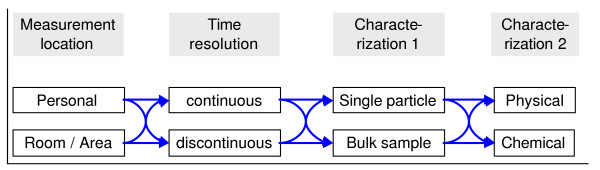
Scheme of particle characterization for exposure assessment.

Figure 4 provides a schematic example how particle measurements can be done in relation to exposure assessment. This scheme clearly indicates the possible approaches to exposure measurements related to Nanomaterials. A thorough review of available techniques and evaluation of this with the toxicological community will be an important tool to outline the future directions in R&D. This should be done with an international expert group.Exposure assessments to NP and their agglomerates can only be done currently by room/area measurements since personal samplers specifically for NP do not exist. Therefore systematic and harmonised measurement strategies must be developed and employed taking into account the special variability on concentrations. Specific care has to be taken in the identification of sources since sources like nearby traffic may significantly influence the concentrations without being related to industrial Nanomaterials.

**Figure 5:**
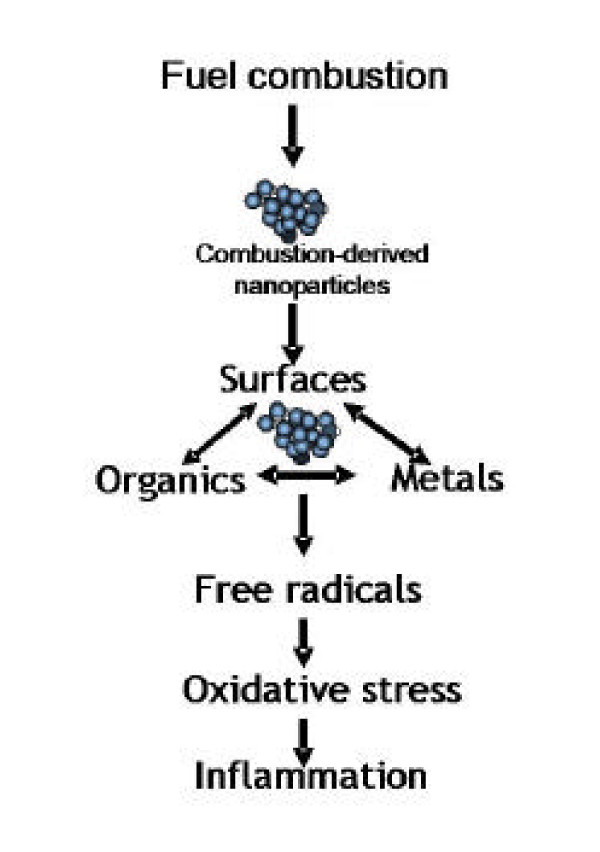
Diagram to illustrate the likely relationship between the three main characteristics of combustion derived NP and their ability to cause inflammation.

Topics generally to be harmonized are:

- Measurements techniques for physical and chemical characterization (including speciation if toxicological relevant e.g. of NP and of their agglomerates).

- Measurement parameters for standard not explorative exposure measurements

- Measurement strategies, including area screening and background influences.

Recommendations are:

- International agreement on particle parameter(s) to be determined in occupational exposure.

- Use of novel or currently not used particle measurement techniques in exposure measurements such as particle reactivity (e.g. ESR measurements) along with epidemiological studies.

- Development of personal samplers to determine personal exposure to NP and their agglomerates (number and surface concentration measurements may be possible in the near future).

- Development of a model describing the dispersion and transformation of NP and their agglomerates in the working environment to a) assess location of maximum exposure and b) to develop/plan safe working environments.

- Development of standard measurement methods and strategies to harmonize exposure data for risk assessment and to enable the development of safety standards.

- Standardisation of number concentration measurements including definition of the lower and upper particle size range determined and defined relative humidity.

### 3.5 Different product lines in view of exposure

NP currently produced in large mass quantities globally include Titanium, Silica, Alumina, Metals and Carbon. Other NPs currently used are include carbon nanotubes and Barium sulfate. Only few studies systematically approaching exposure to NP and their agglomerates exist. A further handicap is that the reports of these studies are often not available for the public. Examples of these studies are given in e.g. Proceedings of the International Symposium "Dusts, fumes and mists in the workplace" [28]. Some limited measurements of particle size distributions and number concentrations were conducted. Kuhlbusch et al. [29] determined number size distributions and mass concentrations in various working environments at several carbon black production plants.

These are just examples, but a much more systematic and comprehensive approach is necessary to derive a good overview of exposure to industrial Nanomaterial and especially Nanoparticles.

Recommendations are:

- Development of a systematic categorization system for Nanomaterials based on physical-chemical information of the Nanomaterial and related to toxicological screening methods.

Systematic compilation of Nanomaterial production processes, handling and products for the assessment of the current knowledge and identification of processes and products with lack of information (may be internally to include non-public studies).

## 4. Toxicology of nanoparticles

### 4.1 Evidence for nanoparticles in ambient particle effects

The largest database on the toxicity of Nanoparticles has originated from the PM_10 _literature, where the 'NP hypothesis' has proved to be a powerful force for research. Therefore we consider it relevant to discuss this evidence in the expectation that it will shed light on the toxicity of engineered NP. The idea that combustion-derived Nanoparticles (NP) are an important component that drives the adverse effects of environmental particulate air pollution or PM_10 _comes from several sources [30]:

1) Much of the mass of PM_10 _is considered to be non-toxic and so there has arisen the idea that there is a component(s) of PM_10 _that actually drives the pro-inflammatory effects and combustion-derived NP seems a likely candidate.

2) NP are the dominant particle type by number suggesting that they may be important and their small size means that they have a large surface area per unit mass. Particle toxicology suggests that, for toxic particles generally, more particle surface equals more toxicity.

3) Substantial toxicological data and limited data from epidemiological sources support the contention that NP in PM_10 _are important drivers of adverse effects.

#### 4.1.1. What effects of PM_10_?

The adverse health effects of PM are measurable as exacerbations of respiratory disease and deaths as well as hospitalisations and deaths from respiratory and cardiovascular disease [31]. Inflammation is the common factor that binds together these adverse effects and the ability of NP to cause inflammation can be seen as an important property and this is addressed fully below. It is not clear what effects of PM10 have pulmonary inflammation as a prerequisite and what effects could potentially be driven by exposures at levels below those causing inflammation. There is also the potential for pulmonary inflammation to results in changes in membrane permeability that in turn may impact the potential for particles to distribute beyond the lung. In addition, some NP are known to be able to redistribute from their portal of entry e.g. interstitialise in the lungs [32] and enter the brain [33] and the blood [34] (see 4.2). Therefore some NP may have the extra potential of affecting cardiovascular disease directly. However, data to date are limited and not all studies of Nanoparticles have shown significant translocationfrom lung to the blood. Understanding the particle characteristics (size, charge, lipophilicity, protein adsorption) that impact the translocation process and the potential for dose rate effects on translocation will be important both in terms of dose rate effects on competing clearance mechanisms (mucociliary) and potentially on barrier function. Beyond that, understanding clearance kinetics of Nanoparticles will also be important in understanding their potential for adverse effects. Therefore NP have the potential of affecting cardiovascular disease both indirectly via pulmonary inflammation and directly through particle distribution although important, this property of redistribution has yet to be demonstrated for NP present in real PM10. Of note, many of the effects attributed to exposure to high levels of ambient particulates are also present in patients with chronic obstructive pulmonary diseases (COPD) (presumably independent of ambient particle exposure status). COPD and other disorders associated with reduced lung function are strong risk factors for cardiovascular events, independent of smoking. While the mechanism(s) for this observation is/are largely unknown, there is evidence that suggests that low-grade, systemic inflammation related to COPD may play an important role. In a severity-dependent fashion, circulating levels of C-reactive protein, fibrinogen, and other inflammatory biomarkers are 1.5 – 3.0 times higher among individuals with COPD than in those without [35] Importantly, COPD patients with elevated C-reactive protein and other inflammatory biomarkers have a higher risk of cardiac events than those with normal C-reactive protein levels. The risk of cardiovascular events may be further amplified by the use of bronchodilators that adversely alter the delicate balance of sympathetic and parasympathetic forces within the autonomic nervous system. In sum, COPD is a risk factor for cardiovascular diseases. Persistent systemic inflammation may, in part, be responsible for this relationship. It is important to keep in mind that the effects of ambient air pollution involve a complex interplay between the complex constituents of the ambient particles and underlying disease. However, it is clearly appropriate to include endpoints reflective of ambient particulate exposure in studies of the effects of engineered Nanoparticles to understand the potential for different types of particles to elicit these effects and the relative dose/response.

#### 4.1.2. Characteristics of NP in PM_10_

NP in PM_10 _are mainly produced as a by-product of combustion and in conurbations predominantly emanate from traffic vehicles. It is possible that manufactured Nanoparticles such as NP carbon black or NP titanium dioxide are locally important, near industrial facilities or dumps, but this is likely to be the exception. Fly ash from the burning of pulverised coal contains a NP fraction [36] and so point sources of this NP may impact on PM_10 _effects locally. Even though combustion of coal and oil also produces NP-sized particles [36,37], in most urban situations, where most exposure occurs, it is the vehicle emission that contributes the majority of the NP [37]. In addition, it needs to be mentioned that secondary UFP do contribute to ambient UFP exposure [38] and that the solubility of these UFP is greater than for primary combustion derived UFP.

In most cases UFP do not occur as single particles but as aggregates of smaller particles. Both diesel and gasoline combustion results in the production of an aerosol that is nanoparticle in primary particle size [39-41]. Kubo et al [42] identified two peaks of Nanoparticles in diesel exhaust – a 30 nm (nuclei) mode comprising volatile species that can be defined as nanodroplets and an 80 nm nanoparticle (accumulation) mode with a solid carbon core. The primary particles can rapidly form aggregates [40,41] and the nanodroplets may volatilise over time and so neither be directly measured as NP by a size-selective sampler. The nanodroplets of organic material have an extremely complex chemical composition, comprising C_13_-C_35 _hydrocarbons (e.g. aldehydes, ketones and alkyl nitrates), PAHs (e.g. benzo(a)pyrene) and nitro-PAHs (e.g. nitropyrene) [41]. The nanodroplets are formed largely from branched alkanes and alkyl-substituted cycloalkanes from unburned fuel and lubricating oil [43], plus a few percent sulphuric acid [44]. Background levels of NP in the outdoor environment are in the range 5000–10,000 particles per cm^3 ^rising, during pollution episodes, to 3,000,000 particles/cm^3 ^[45]. However NP readily aggregate into larger size classes [46], and these aggregates may not be detected as NP but it is known that aggregates retain the toxicity of the NP that comprise them (see 4.1.3). NP are also readily measurable indoors and since we spend more than 90% of our time indoors then the indoor environment can be important and it is notable that vacuum cleaning and cooking have been reported to dramatically increase airborne NP numbers [47,48].

#### 4.1.3. Epidemiological evidence of a role for NP in the adverse effects of PM_10_

There is only indirect epidemiological evidence that combustion NP are a factor in PM_10 _that drives the adverse health effects. More data are likely to be available soon as up to this point there have been few long-term measurement campaigns that measured NPs. Most epidemiological studies cannot discriminate whether effects are due to NP or not as 1) they do not measure the NP; 2) NP in aggregates or adhered to larger particles may be registered by a sampler as larger-than-NP in size. However we know from controlled toxicological studies that aggregates retain the essential extra toxicity of NP; aggregates do not behave like a geometric particle the size of the aggregate because the greater surface area of the individual particles is retained in the aggregate and is involved in toxicity.

Peters et al showed a correlation between the number of ultrafine particles (NP) and decreases in evening peak flow in a panel of 27 non-smoking asthmatics [49]. Maynard and Maynard [50] used a historical approach and reinterpreted exposure data on the London smogs. They recalculated exposure as surface area, a manoeuvre that takes particle size into account and emphasises the role of combustion-derived NP surfaces and particle number. Much of the London smog particulate in historic lungs can be seen to be NP and generally supports the 'ultrafine' or NP hypothesis [51]. The recalculation of Maynard and Maynard showed that surface area was a better indicator of heath effects associated with exposure to PM. Such a finding supports the contention that NP are important because they have such a high surface area per unit mass compared to bigger particles. Peters et al demonstrated two different time-response correlations between PM levels and myocardial infarction- one at 2 hours and one at 24 hours [52]. NP have been reported to rapidly interstitialise and become bloodborne following instillation [34] and inhalation [53], although translocation to blood and tissues in the latter study was only minor. A direct effect of bloodborne particles on atheromatous plaques could explain the 2 hour relationship reported by Peters [54] whilst a more slowly developing inflammatory response affecting plaques could explain the 24 hour effect. These are, of course speculative conclusions based only on correlations.

#### 4.1.4. Toxicological data supporting a role for NP in the adverse effects of PM_10_

It should be noted that there are several mechanisms whereby NP could lead to inflammatory effects, as is the case for larger particles. These mechanisms could be based on the large surface area of particle core or on soluble components released by the NP. Several toxicological studies support the contention that NP in PM_10 _could drive inflammatory effects. There are a number of components of PM_10 _that contribute to the mass but have little toxicity – these include salts such as sulphates, chlorides and ammonium salts and nitrates, but also wind-blown or crustal dust. In fact within PM_10 _there are only few components that toxicologists would identify as likely mediators of adverse effects – i.e. particle surfaces, organics, metals and endotoxin (in some PM samples). In fact, a large surface area, organics and metals are all characteristic of combustion-derived particles [55] and so these have attracted considerable toxicological attention [56], [57,58]. Diesel exhaust particles (DEP) cause inflammation in the lungs in subjects exposed to it for a few hours [59,60] and in animals [61] and causes pro-inflammatory effects in cells in culture [62]. It is difficult to untangle, in a combustion particle sample, the relative roles of surface, organics and metals, although this has been most attempted *in vitro *(see below).

#### 4.1.5. NP surfaces

Combustion nanoparticles with very low organic and metal content- e.g.NP carbon black – have been investigated and have been shown to cause inflammation via their surface characteristics and not by any soluble material [63]. In support of this, a wide variety of low toxicity, low solubility particle types, not all combustion derived, were shown to induce inflammation in direct relation to their surface area [16]. From this it is clear that a high surface area of particles in the lung is sufficient to initiate inflammation.

#### 4.1.6. NP-associated organics

Organic chemicals associated with NP play a role in the pro-inflammatory effects of diesel exhaust particles, as demonstrated in a number of in vitro studies. However it should be noted that the types of studies described below, where done with organics extracted from diesel or carbon black particles and then used on cells. Those studies do not take into account bioavailability from these organics in particles, and often result in doses of organics that are enormous compared to what could anticipated from a plausible diesel particle exposure [64].

DEP caused modest stimulation of different cytokines and growth factors, including interleukin-8 (IL-8), granulocyte macrophage colony-stimulating factor (GM-CSF) by epithelial cells and this activity was lost upon extraction of the organic matter [65]; the benzene extracts were found to contain most of the stimulatory activity seen in the whole DEP. Benzene extracts contained almost 90% of the B(a)P content and the authors concluded that PAHs such as B(a)P were likely responsible for the stimulation of cytokine production by the epithelial cells. Boland *et al *[66] demonstrated that DEPs stimulated IL-8, GM-CSF, and IL-1β release from the bronchial epithelial cell-line 16HBE. Furthermore they contended that this was related to the amount of adsorbed organic compounds, because, in comparison carbon black with virtually no adsorbed organic matter, did not cause cytokine release. In support of this the exhaust gas post-treatments, which diminished the adsorbed organic compounds, also reduced the DEP-induced increase in GM-CSF release. Further studies with the organic extracts confirmed that most of the stimulatory activity seen in the DEP sample was in the organic fraction [67]. In another study PAH extracted from DEP induced expression of IL-8 and RANTES in peripheral blood mononuclear cells [68], demonstrating that both macrophages and epithelial cells could be important in the pro-inflammatory effect induced by DEP in the lungs. Chin et al [69] demonstrated that carbon black-treatment of the RAW264.7 mouse macrophage cell line had no effect on TNFα release but that the addition of BaP to the particles caused them to become stimulatory for TNFα.

#### 4.1.7 NP-associated metals

Metals have been reported to be involved in the oxidative stress produced by diesel soot [70] and transition metals have been reported to be involved in the redox cycling of quinones, a major organic species considered to be involved in oxidative stress caused by ambient particles [71,72]. Experimentally, transition metals and NP surfaces act synergistically in producing inflammation [73], and the final common pathway of oxidative stress mediated cytokine gene transcription seems the likely explanation [74].

### 4.1 Summary

It is generally considered that PM_10 _mass is not the true driver of the adverse effects of particulate air pollution and toxicological considerations of the components suggest that one or more components in fact drives these effects and that PM_10 _mass is really a surrogate. There is both direct and indirect evidence that combustion-derived NP is one important component that could be a key exposure in the adverse effects of PM_10_. Three important pro-inflammatory factors are associated with combustion-derived NP including high particle surface area, organics and metals (Figure 4.1). This 'nanoparticle hypothesis' needs to be further tested in epidemiological and toxicological scenarios. It remains to be investigated whether and how relevant these reports of adverse effects of environmental combustion-derived NP are for engineered Nanoparticles, which may dramatically differ from them in physical and chemical characteristics.

### 4.2. Distribution and kinetics of nanoparticles in the body

#### 4.2.1. Deposition in the respiratory tract

Nanoparticle deposition in the respiratory tract is determined predominantly by diffusional motion due to thermal motion of air molecules distorting particles from their stream lines of the inhaled and exhaled air towards the walls where they deposit. Diffusional motion is affecting particle deposition through three important components (aerosol properties and physiology) during breathing:

(a) particle dynamics including the size and shape and its possible dynamic change during breathing;

(b) geometry of the branching airways and the alveolar structures; and

(c) breathing pattern determining the airflow velocity and the residence time in the respiratory tract and including nose versus mouth breathing.

**Figure 6:**
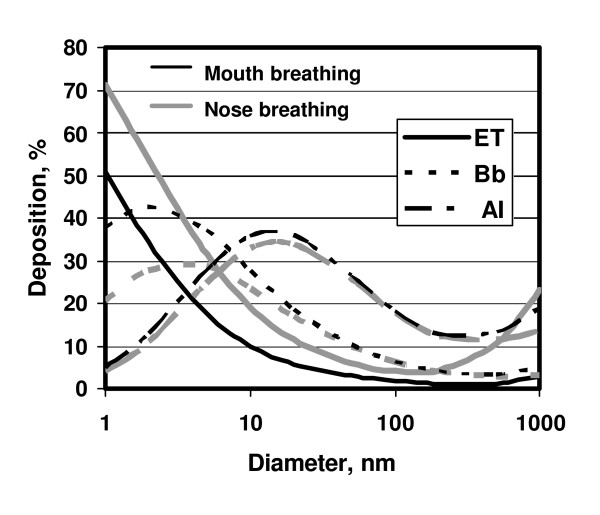
Regional deposition of inhaled NP with diameters between 1 nm and 1000 nm for nose and for mouth breathing in the extrathoracic airways (ET), the bronchial airways (Bb) and the alveolar region (AI) during breathing at rest, as predicted by ICRP 66 model (ICRP, 1994).

Regarding regional particle deposition the respiratory tract acts as a series of filters starting with the nose or mouth, via the various diameters of airways to the alveoli. Fig 6 displays that particles of different sizes deposit differently in the airways, as well as the alveolar region. The smaller the particle, the higher the probability that a particle will hit the epithelium of a lung structure. The deposition increases in all regions of the lung with decreasing particle diameter below 500 nm due to the increasing diffusion mobility. This means that Nanoparticles of different sizes can have different effects in different parts of the lungs. This may be particularly important in children with developing lungs and in asthma and COPD patients. These diseases may also cause increased deposition of PM in diseased parts of the lungs up to several fold, which may deteriorate their functions.

**Figure 7:**
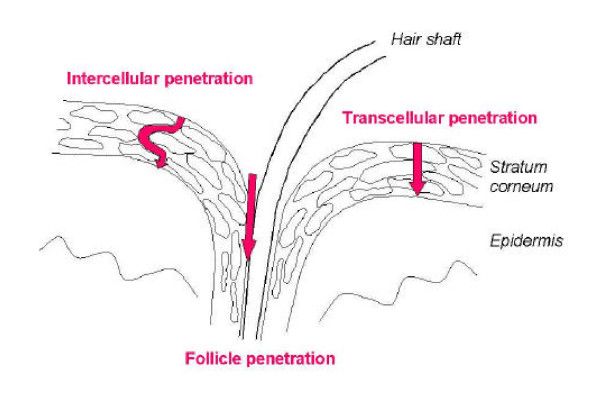
Penetration pathways of topically applied substances through the skin.

#### 4.2.2 Fate of particles in the lungs

On the walls (epithelium) of the respiratory tract particles contact first the mucous or serous lining fluid and its surfactant layer on top. Therefore, the fate of particle compounds soluble in this lining fluid need to be distinguished from slowly dissolving or even insoluble compounds.

*Slowly dissolving and insoluble NP deposited on the airway wall *will only be partly moved by action of ciliated cells with the mucus or by cough within 1–2 days to the throat (larynx), where they are swallowed. The smaller NP are the more they are retained in and beyond the airway epithelium.

*Slowly dissolving and insoluble NP deposited in the alveolar region *will only be taken up and digested to a limited amount by specialised defence cells that are called macrophages and are located in the alveoli. Therefore, alveolar macrophages will determine the fate of this rather small fraction of NP. The remaining NP will interact with the cells of the epithelium such that NP will be taken up by those cells or transported into the interstitial spaces. As a result most NP will be no longer retained as free particles on the epithelium.

As a result insoluble NP may stay for months and years in the lungs. The most prominent clearance pathway out of the alveolar region is NP transport by alveolar macrophages to the larynx where they will be swallowed and excreted after passage through the gastro-intestinal tract). In addition, current discussion focuses on another transport function of the vesicular caveolae that transport from the luminal to the mucosal side of epithelial and endothelial cells. Transport within caveolae for macromolecules with molecular radii of several nanometres seems to exist across the alveolar-capillary barrier as a pathway for protein delivery from lung to blood. This might be another mechanism for solid UFP transport, given that the openings of the caveolae range between 0.04 and 0.1 μm.

### Translocation from lung to the blood

Conflicting studies have been reported regarding particle translocation after inhalation of NP in the lung, reviewed recently by Borm & Kreyling [75]. Oberdörster and co-workers observed rapid translocation towards the liver of more than 50% of ^13^C-labeled NP (26 nm size) within 24 hours in a rat model [76]. Kreyling and co-workers, however, observed only minute (< 1 %) translocation of iridium NP (15–20 nm size) into the blood of rats reaching not only liver but also spleen, kidneys, brain and heart [53]. Conflicting results in human studies are also reported. Nemmar et al [34] demonstrated a rapid 3 – 5 % uptake of radiolabeled carbonaceous NP into the bloodstream within minutes of exposure and subsequent uptake in the liver. In contrast, neither Brown et al [77] nor Mills et al [78] could find any detectable particulates (<1% of inhaled NP, limit of detection) beyond the lungs and cleared fractions via airways and gastro-intestinal tract using similar carbonaceous NP as Nemmar et al. However, Nemmar and co-workers demonstrated in their hamster model, the importance of surface properties like charge since polar surface showed different translocation rates across the respiratory epithelium into circulation [34].

#### 4.2.3. Uptake in the Gastrointestinal tract (GI-tract)

Nanoparticles and microparticles (0.1–3 um) are ingested at high levels per person per day and it is estimated that 10^12^-10^14 ^microparticles are ingested per person per day in the Western world [79], and concerns mainly silicates and titanium dioxide. They are scavenged by M-cells overlying the intestinal mucosa and in this way circumvent active uptake by intestinal epithelium. A GI route of translocation of ingested ultrafine particles to the blood, is supported by studies in rats and humans that have shown that TiO_2 _particles (150–500 nm) taken in via food can translocate to the blood and are taken up by liver and spleen [80,81]. Furthermore, earlier studies by Volkheimer [82] described a mechanism of persorption in epithelial cells of the GI tract by which even larger particles are taken up into lymphatic and blood circulation and translocate to the liver and other organs. Recently, nanocrystals have become the subject of intense investigation for oral administration of drugs and functional food components. Drugs or food constituents are produced in 100 % pure form in nanocrystals, by precipitation or other processes [83]. Since they prove to be very efficient in vivo, and easy to produce their production for oral application is expected to increase considerably.

In contrast, the studies by Kanapilly and Diel [84] and by Kreyling et al. [53] with ultrafine radioactive metal particles did not show significant translocation from the GI tract to other organs via the blood circulation; nor do these studies show significant translocation to extrapulmonary organs of ultrafine metal particles deposited in the lung. The latter study [53] was done with ultrafine ^192^Ir particles and soluble ^192^IrCl_3 _after administration by gavage in comparison to inhalation or intravenous injection. Phosphate-buffered saline suspensions of ultrafine ^192^Ir particles (5 kBq, 0.2 ml) were administered into the oesophagus of 8 rats. In all of the subsequent biokinetic studies complete faecal and urinary excretion was collected separately. At given time intervals rats were sacrificed, dissected, and a complete balance of ^192^Ir activity in all organs, tissues, blood samples, and excreta was measured gamma spectroscopically. Rats were anesthetized with isoflurane as already described. After oesophageal administration of ultrafine 192Ir particle suspension, virtually the whole amount of ^192^Ir was found in faecal excretion within 2–3 d. During the 6-d observation period no detectable ^192^Ir in urine was observed at any day. Six days after administration there was no detectable ^192^Ir in any organ or tissue of the body. Hence, it was concluded that for these particles there was no uptake and/or absorption from the GI tract. It needs to be mentioned however, that for the same particles (18 nm ^192^Ir) also virtually no pulmonary translocation took place.

#### 4.2.4 Dermal uptake

In principle there are three possible penetration pathways of topically applied substances through the skin: the intercellular penetration, the intracellular penetration, and the follicular penetration.

In the past, the penetration processes were described as a diffusion through the lipid layers of the stratum corneum [85]. Liposomes with a diameter between 20 nm and 200 nm were found to be active carriers of topically applied drugs into the living epidermis via the intercellular penetration route [86]. Initially, it was presumed that the follicular penetration does not play a dominating role in this process, because the amount of hair follicles of the total skin surface was estimated to be not more than 0.1% of the total skin surface area [87]. However, several in vitro and in vivo studies suggested a potential role of follicular penetration in the dermal penetration process. Feldman [88] and Maibach [89] observed regional variations of percutaneous absorption in different skin areas. They assumed that the density and size of hair follicles might be the reason for their findings. Hair follicle mediated uptake is further discussed in the paragraph on dermal effects of NP.

**Figure 8:**
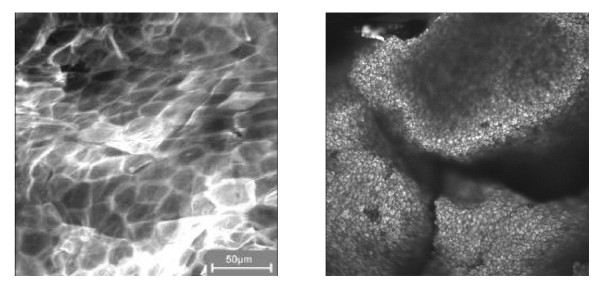
In vivo investigation of the distribution of a formulation containing ethanol (left picture) and Nanoparticles (right picture) on the surface of human skin.

The on-line, *in vivo *investigation of the distribution of topically applied formulations containing Nanoparticles on the skin surface became possible by the use of fibre-based laser scanning microscopes. In this case the optical imaging and scanning system is incorporated into a hand piece, which can be applied to any skin area [90]. A skin surface treated with a formulation containing ethanol and a formulation containing Nanoparticles is presented in Figure 8. In the first case, the corneocytes of the skin surface were easy to recognize. When a formulation containing NSP was applied, a thin protection film covers the skin surface.

### Dermal uptake of NP through hair follicles

As previously mentioned, dermal absorption/penetration processes have been described as a diffusion process through the lipid layers of the stratum corneum (4. x) Follicular penetration was previously believed to play a minor role in percutaneous penetration, given that hair follicles were estimated to occupy less than 0.1% of the total skin surface area. However, several studies suggested that follicular penetration might contribute to the overall penetration rate. For example, Feldman [88] and Maibach [89] found regional variations of percutaneous absorption in different body areas and assumed that, in addition to skin thickness, the density and size of hair follicles contribute to the differences in penetration rates.

A recent observation of cadmium sulphide particle deposition in follicles [87] prompted further investigations on follicular penetration of solid particles. The results suggested that, after application of a fine material of a diameter of less than 1μm in an appropriate vehicle, the material is mainly found in the upper parts of the horny layer as well as in follicular orifices. A material with a particle diameter of 3 to 10 μm was observed in the follicle orifices only, whereas material of more than 10 μm in diameter also remained on the skin surface. This observation was made for several materials, including adapalene (a synthetic retinoid) crystals, polystyrene beads, aniline dansylate as well as benzoyl peroxide. It is conceivable that from this follicular reservoir soluble compounds may further diffuse into the viable layers of the skin.

**Figure 9:**
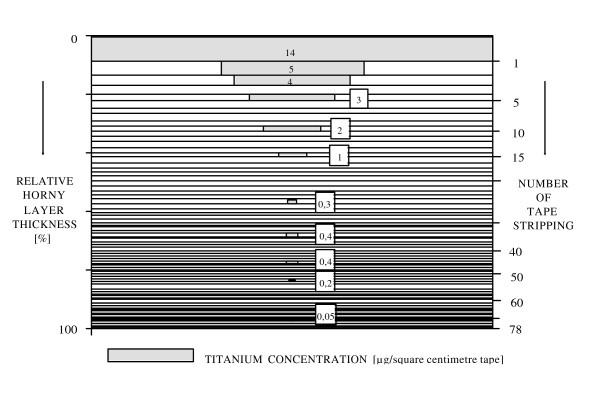
Penetration of NP-size coated titanium dioxide into the horny layer 1 hour after long-term sunscreen application.

Lademann et al. [91] investigated the penetration of coated titanium dioxide NP into the stratum corneum of living human skin by tape stripping and biopsies in combination with spectroscopic measurements. After application of a sunscreen containing TiO_2 _NP, the largest amount of coated titanium dioxide was localized in the upper part of the stratum corneum, although minimal amounts of TiO_2 _could be detected on tapes obtained at the end of the stripping procedure as shown in figure 9.

**Figure 10:**
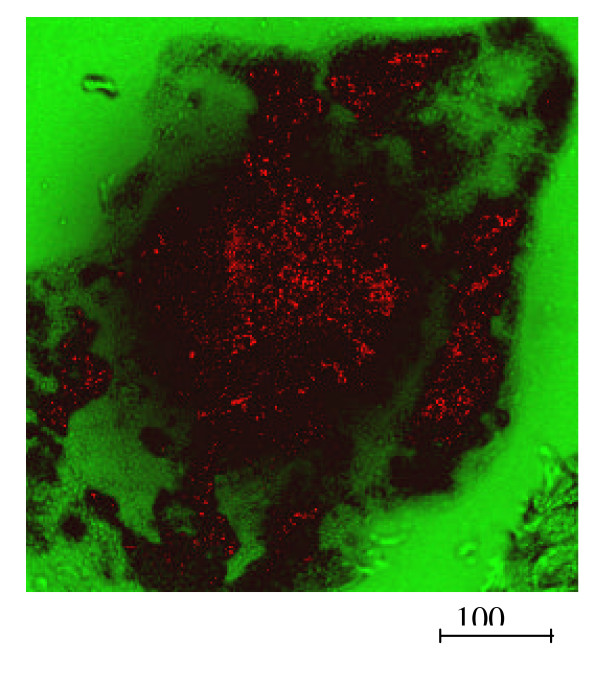
Superposition of the transmission and fluorescence image of a pilosebaceous orifice on a 25th removed tape strip stained with OsO_4 _obtained by laser scanning microscopy, the distribution of titanium dioxide coating inside the mark of a pilosebaceous orifice is seen as red spots.

Small white spots of sunscreen material could be observed visually in some follicle orifices after removing several strips from the treated skin surface, suggesting that TiO_2 _was present in the orifices. In order to investigate the concentration of TiO_2 _in follicles, the distribution of the TiO_2 _NP on tape strips from different parts of the stratum corneum was analysed after staining with OsO_4 _[91]. Presence of TiO_2 _in pilosebaceous orifices could be shown on tape strips from the lower parts of the stratum corneum. The coating material of the TiO_2 _Nanoparticles on the tape strips emitted a typical fluorescence, which allowed the detection of Nanoparticles in stained areas of the orifices by laser scanning microscopy (figure 10).

On corneocytes outside the follicle channels or adhesive film from areas not covered with corneocytes, no fluorescence was detected. Skin biopsies were collected in order to localise TiO_2 _in deeper parts of the epidermis. Three characteristic areas could be distinguished by X-ray fluorescence microscopy, i.e.:

• Stratum corneum: presence of titanium dioxide NP

• Interfollicular epidermal tissue below the stratum corneum: absence of titanium dioxide NP

• Follicles: in approximately 10% of follicles fluorescence was observed suggesting presence of titanium dioxide The titanium concentration decreased in the lower regions of these follicles. Detected TiO_2 _concentrations inside the follicles were by two orders of magnitude lower than those in the upper part of the horny layer.

A penetration of Nanoparticles through the horny layer could not be detected by X-ray fluorescence. Overall, even these highly sensitive methods failed to detect a penetration of titanium dioxide particles into living tissue of human skin [91]. These data also confirmed the results of an earlier study, which detected no percutaneous absorption of particles in skin samples from humans treated with a microfine titanium oxide-containing sunscreen [92]. A more recent study on the percutaneous penetration of two different micronised titanium dioxide preparations used in sunscreens (a) particle size of 10 to 15 nm, which aggregated to particles of approximately 100 nm, and b) particle size of approximately 100 nm) revealed that these particles remained on the surface of the stratum corneum, and did not penetrate into the stratum corneum or living compartments of the skin [93]. The absence of skin penetration of NP is also consistent with the results of a recent study that measured in vitro the percutaneous penetration of micronised zinc oxide (mean particle size: 40 nm) through pig skin. The study found no measurable penetration of zinc oxide NP into the viable parts of the skin (BASF Study No 52H0546/032193, 2004, unpublished). Although it cannot be excluded that the physical properties of NP may enhance the absorption/penetration of certain substances applied to the skin, such as reported for methanol or octanol [94], the results of available studies suggest that, although small particles may be deposited on the follicle orifice, they do not penetrate the skin via the follicle. This was confirmed by the results of a recent study, which showed that although polystyrene NP (20 to 200 nm) accumulated in the follicle orifices, the particles did not penetrate into the skin or the follicle [95].

#### 4.2.5 Uptake in the Central Nervous System (CNS)

NP may be taken up directly into the brain by trans-synaptic transport, as pointed out in recent publications [33] Such a mechanism was first reported in 1947 for 0.03-μm polio virus in monkeys and was later described for nasally deposited colloidal 0.05-μm gold particles moving into the olfactory bulb of squirrel monkeys. Carbonaceous NP may translocate along the same pathway to the central nervous system (CNS), based on their presence in the olfactory bulb of rats after inhalation [33]. These authors suggest that Nanoparticles gain access from the olfactory epithelium to the olfactory lobe via the olfactory nerves. The olfactory epithelium, a 2.5 cm^2 ^patch in the human nasal passage, is a ciliated pseudo stratified columnar type containing few or no goblet cells. Three cell types are present, the olfactory cell, support cell and basal cell of which only the olfactory cell is chemoreceptive. The olfactory cells are actually quasi-neurones, the axons of which bundle together to make the olfactory nerve leading to the olfactory lobe. The olfactory lobe is a projection of the lower anterior portion of each cerebral hemisphere, which is responsible for processing the nerve impulses relating to the sensation of smell. Neurones are commonly labelled using the retrograde transport along axons of large molecules such as HRP, suggesting that these cells may have the capacity to transport Nanoparticles. In fact single particle tracking has been used to study the transport of glutamate receptors in neurones.

Access of Nanoparticles to neural tissue via the blood brain barrier (BBB) is also possible. Of all the endothelial barriers within the body, the BBB is the tightest, containing specialised tight junctional proteins to minimise paracellular transport. Unless the BBB is compromised the main route of access to the brain for all molecules (except small lipophillic substances such as alcohol) is via a transcellular route, usually involving specific transport proteins. Studies by [96] suggest the presence of a physiological barrier at the basal lamina, analogous to the podocyte in the kidney, that is distal to the anatomic tight junction of the BBB. Using magnetic Nanoparticles and MRI to image their distribution, these studies suggest that the physiological barrier may limit the distribution of some proteins and viral particles after transvascular delivery to the brain, suggesting that the healthy BBB contains defence mechanisms protecting it from blood borne nanoparticle exposure. A number of pathologies, including hypertension and allergic encephalomyelitis, however have been associated with increased permeability of the BBB to Nanoparticles in experimental set ups.

#### 4.2.6 Chronic secondary delivery of NP

Some studies[97,98] described a very small but detectable fraction of NP (^192^Ir) that translocated to secondary target organs like the liver, spleen, brain, and kidneys of about 0.002 each. For these extrapulmonary organs, a peak was found at day 7 after inhalation. Long-term retention data showed no further accumulation, but a net clearance from these target organs was found with time and decreased to close to the limit of detection. As shown in the previous investigation [99] the iridium UFP were virtually insoluble. To distinguish between the soluble and particulate fraction, they performed the same analysis as previously and they were able to detect a particulate fraction which decreased with time [99].

#### 4.2.7. Protein absorption and uptake of NP

Intensive studies had been performed on the systemic translocation of 1- to 4-nm actinide oxide particles from the rat lungs after instillation into the trachea [100,101]. Rapid systemic 24-h translocation of particles bound to either a complex pattern of proteins and/or components of surfactant was observed depending on parameters of particle material and surface properties like the net charge of the particles. The authors clearly emphasize the need to distinguish between absorption of dissolved particle material chelated by citrate anions and proteins like transferrin and particles bound to surfactant components and a protein pattern, which they were unable to resolve. They discuss epithelial pores in the size range of 1 nm as a potential pathway. Besides those pathways, active transport of particle-protein complexes also needs to be considered.

Engineered Nanoparticles have also been developed as devices to deliver drugs and proteins across the GI-tract and liver, to prevent complete degradation (review: Duncan, 2004) [102]. This is related to the fact that Nanoparticles with their large surface will bind or adsorb proteins, which may then function as receptor-agonists [103] or adsorption may protect the protein from recognition by digestive enzymes. The ability of NP to adsorb proteins is dependent on the particle coating [83]. On the other hand after release of the carrier protein, NP can interact with endogenous proteins at the site of deposition. NP below 40 nm have a size comparable to large proteins, therefore leading to the following hypotheses:

• Depending on NP surface properties, NP may complex different endogenous proteins

• Different NP-protein-complexes may have different biokinetics including translocation across membranes

• Endogenous proteins of these complexes may have a different activity or even different function.

**Figure 11:**
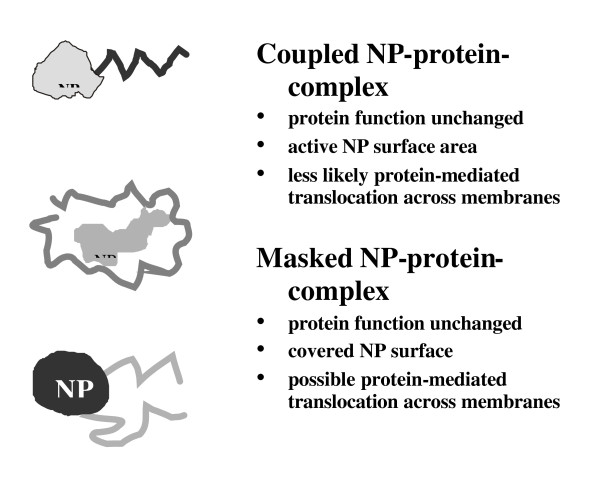
Schematic presentation of potential interactions between Nanoparticles and proteins. The first example shows the intended (covalent) binding of a protein to an NP as a drug-delivery-tool. The second example shows how proteins may absorb on the NP surface, thereby masking the particle properties and loosing functional protein. The third example shows how NP can bind and breakdown proteins, through their active surface area [15].

Preliminary studies using different ultrafine particles indicate that these bind differently to a number of proteins in native rat broncho-alveolar lavage fluid (Kreyling, personal communication). If these studies are confirmed the biokinetics of different NP-protein complexes could provide the basic explanation for the different observed translocation patterns described by the various studies as reviewed in chapter 5. At the same time functional changes in proteins in such complexes may be another mechanism by which particularly small NP with their large surface area as a binding interface may induce protein mal-functioning which may lead to the pathogenesis and adverse health effects. Figure 11 illustrates some of the theoretical possible scenarios.

#### 4.2 8 Elimination and kinetics of NP

On reaching the blood, Nanoparticles may be eliminated by different mechanisms which are dependent on the route of absorption and their surface properties. For example, absorption via the lung, skin or gut could be as particles contained within phagocytic cells such as macrophages, as free single or aggregated particles, or particles associated with serum proteins. Free single or aggregated particles are likely to be removed from the circulation via phagocytic cells in the reticuloendothelial system and hence will accumulate in organs such as the liver. It is unlikely that Nanoparticles will remain as discrete single particles in the body since they are likely to be coated very quickly in biological molecules such as proteins. Nanoparticles contained within i.v. preparations are likely to be formulated to allow targeting to specific compartments within blood and accumulation within specific target organs or tissues such as tumours. This targeting may be achieved through surface modification of the Nanoparticles.

In terms of toxicology and pharmacology, **metabolism **usually refers to the chemical alteration of a xenobiotic via enzymatic mechanisms. The chemical alteration of inorganic Nanoparticles, such as TiO_2 _is unlikely, although any chemical groups added to the particle surface could be modified enzymatically or non-enzymatically within the body. As for polymers, the enzymatic alteration of these substances is likely to be very specific to the polymer composition and characteristics. Fullerenes have been shown to be metabolized in aquatic systems and it is therefore assumed that carbonaceous NP especially those with branched side chains or hydrophilic groups are targets for normal human metabolic machinery, which is driven by oxidative enzymes [104].

The most common route of **excretion **for xenobiotics is via the kidneys into urine. This process involves filtration of the blood through a complex filtration membrane in the glomerulus of kidney nephrons. The size of the fenestrae within the glomerular filter is limited to allow the passage of small molecular weight substances whilst preventing the filtration of larger molecules such as proteins. For substances up to a molecular weight of 7,000 Dalton the filter allows free filtration, above 7,000 D filtration is retarded, and above 70,000 D filtration is insignificant. Poly(amidoamine) (PAMAM) dendrimers, 5 nm in diameter have been shown in a mouse model to be excreted via urine with accumulation in kidney observed [105]. Some studies have suggested that drugs can be targeted to the kidney by incorporation in Nanoparticles to facilitate uptake by glomerular essential cells [106,107], however the short or long-term impact of accumulation within the filtration apparatus on renal function has not yet been addressed. It is conceivable that particles of an appropriate size could easily block the renal fenestrae leading to rapid kidney failure and death. Unpublished observations by some research groups suggest that this is a realistic process (Curtis, personal communication). Surface or charge modifications of the Nanoparticles may help to prevent association of the particles with the filtration membrane.

Excretion of xenobiotics from the body also occurs via bile, produced by liver parenchymal cells (hepatocytes) and secreted into bile ducts before delivery into the gastrointestinal tract for excretion in faeces. After oral administration of polymethyl [1-^14^C]methacrylate Nanoparticles to rats [108] radioactivity was detected in bile and urine, although it was not confirmed that this radioactivity remained particle bound. Large molecular weight substances such as horse radish peroxidase (HRP) are known to gain access to the bile via transcytosis mediated by vesicular transport [109]. It is conceivable that the same route of transport could be available to Nanoparticles. The studies by Lowe *et al*., [109] also indicate that liver damage increases the likelihood of paracellular routes of entry for HRP into bile.

### 4.2. Summary

After inhalation, oral administration or parenteral administration NP can get access to the lung, gastrointestinal tract and brain. Dermal exposure and uptake is being debated as an important uptake route for NP, since NP are present in many cosmetic products as vehicles for moisturizers or in shampoos, detergents or sunscreens. Up to now, dermal uptake of Nanoparticles has not been demonstrated beyond the submucosa. No studies have been conducted to address the question whether Nanoparticles topically applied to human skin can reach the dermal compartment and via that route enter the circulation. Given the high bioavailability of Nanoparticles, however, these studies are of obvious importance, in particular with regard to individuals with a skin barrier defect such as atopics.

The uptake and transport mechanisms in the lung and GI-tract differ qualitatively and quantitatively from fine particles. Transport within caveolae for macromolecules with molecular radii of several nanometres seems to exist across many barriers as a pathway for protein delivery from lung to blood [110]. This might be another mechanism for solid NP transport, given that the openings of the caveolae range between 0.04 and 0.1 μm.

Non-soluble Nanoparticles can stay for years in the lungs, GI-tract or brain; they are less well taken up by professional macrophages of the defence system but interact with cells of the epithelium, the interstitial tissue and vascular cells allowing pro-inflammatory reactions of these cells which usually do not see any particles. In addition, Nanoparticles can bind to proteins or translocate into the circulation and reach secondary target organs like liver, spleen, kidneys, heart and brain; rates and fractions are still under debate and depend particularly on the chemical and surface properties of Nanoparticles.

### 4.3 Effects of nanoparticles

#### 4.3.1. Pulmonary effects

Pulmonary toxicity studies in rats demonstrate that ultrafine particles (generally synonymous with the term "Nanoparticles" (see Chapter 1) produce enhanced inflammatory responses when compared to larger-sized particles of identical chemical composition at equivalent mass concentrations [111,112]. Surface area and particle number determinations appear to play important roles in ultrafine particle lung toxicity. Contributing to the effects of ultrafine particle toxicity is their very high size-specific deposition rate when inhaled experimentally as singlet ultrafine particles rather than as aggregated particles. Some evidence suggests that inhaled ultrafine particles, after deposition in the lung, largely escape alveolar macrophage surveillance and gain greater access to the pulmonary interstitium through translocation from alveolar spaces through epithelium [111,112].

A limited number of studies have been reported which have assessed the inhalation toxicity of ultrafine particles by laboratory animals at very high particle concentrations. Some hazard-based toxicity studies were conducted to investigate pulmonary effects caused by lung particle overload, i.e., induction of lung tumours in rats at high retained particulate lung burdens, Specifically, chronic inhalation studies with ultrafine (P-25) or fine-sized TiO_2 _particles (average primary particle sizes ~ 20 nm and ~ 270 nm, respectively) have shown that less than one-tenth the inhaled mass concentrations of the aggregated ultrafine particles, compared with the fine particles, produced equivalent numbers of lung tumours in rats in these 2-year studies (approximately 16–30%) [113,114]. In addition, shorter-term pulmonary toxicity studies with ultrafine and fine carbon black, nickel, as well as TiO_2 _particles in rats [115-117], have demonstrated enhanced lung inflammatory potency of the ultrafine particles when compared to fine-sized particulates of similar composition. When the instilled doses were expressed in terms of particle surface area, the responses of the ultrafine and fine TiO_2 _particles fell on the same dose-response curve. This is because a given mass of ultrafine particles has a much greater surface area when compared to the same mass of fine-sized particles and therefore is more likely to cause particle overload in the lung. Thus, from a toxicological and regulatory viewpoint, it will be important to delineate the pulmonary effects of ultrafine particles in rats at overload vs. non-overload conditions.

##### Systematic comparison of fine and ultrafine particles

It may be surprising to note that the toxicity database for systematic comparisons of the pulmonary effects of ultrafine/Nanoparticles vs. fine-sized particles in rats is sparse- and consists of studies on only two particle-types: namely titanium dioxide and carbon black particles [75]. Moreover, the rat model, for which most if not all of the nano vs. fine size comparisons have been reported, is known to be an extremely sensitive species for developing adverse lung responses to particles, particularly at overload concentrations. As a consequence, chronic (two-year), high-dose, inhalation exposures in rats with poorly soluble, low toxicity dusts can ultimately produce pulmonary fibrosis and lung tumours via an "overload" mechanism. The tumour-related effects are unique to rats and have not been reported in other particle-exposed, rodent species such as mice or hamsters, under similar chronic conditions. It has been postulated that the particle-overload effects in rats result in the development of "exaggerated" lung responses, characterized by increased and persistent levels of pulmonary inflammation, failed clearance, cellular proliferation, fibro-proliferative effects, and inflammatory-derived mutagenesis, and this ultimately results in the development of lung tumours.

##### Role of surface chemistry

To complicate further the issue of nanoparticle toxicity, the results of recent pulmonary bioassay studies in rats suggest that, on a mass basis, not all nanoparticle-types are more toxic than fine-sized particles of similar chemical composition. As mentioned previously, the limited numbers of studies that have been reported suggest that ultrafine (P-25) TiO_2_particles produced greater pulmonary inflammation when compared with fine-sized TiO_2 _particles. However, in contrast to the conclusions of the earlier studies with P-25 type ultrafine TiO_2 _particles, the results of recent preliminary studies comparing the effects of nano- vs. fine-sized particles, have indicated that pulmonary exposures in rats to uncoated TiO_2 _nanoscale rods (200 nm lengths × 30 nm diameters) and TiO_2 _nanoscale dots (particle size < 30 nm) did not produce enhanced lung inflammation in rats when compared to fine-sized TiO_2 _particle exposures (particle size ~ 270 nm). In general hydrophobic TiO_2 _seems to be less inflammatory than naive TiO_2_, regardless of particle size [118,119].

Using a similar pulmonary bioassay protocol, lung bioassay studies have compared the toxicity effects in rats of uncoated nanoscale quartz particles (50 nm) vs. fine-sized quartz particles (particle size ~ 1600 nm). Accordingly, at equivalent mass doses, the nanoquartz particles produced less intensive and sustained pulmonary inflammatory and cytotoxic responses when compared to the effects produced by the Min-U-Sil quartz particles [120]. These preliminary findings are intriguing since crystalline quartz silica particles are classified as a Category 1 human carcinogen by the International Agency for Research on Cancer (IARC)[40]. However, also recent work with surface modified fine quartz has demonstrated that both acute and chronic inflammation and genotoxicity [121] is inhibited.

A number of factors are likely to influence the pulmonary toxicity of Nanoparticles. These include:

1) Particle number and size

2) Surface dose

3) Surface coatings on particles, particularly for engineered nanoparticulates [122];

4) The degree to which ambient ultrafine particles "age" and become aggregates, or engineered Nanoparticles aggregate/agglomerate, due in large part to surface characteristics;

5) Surface charges on particles, as well as particle shape and/or electrostatic attraction potential (as is the case for engineered single-wall carbon nanotubes- which readily agglomerate [123].

6) Method of particle synthesis (i.e., whether formed by gas phase (fumed) or liquid phase (colloidal/precipitated) synthesis and post-synthetic modifications.

The degree to which engineered Nanoparticles aggregate in the ambient aerosol or occupational environment and subsequently do or do not disaggregate following inhalation and particle deposition in the lung will strongly influence particle deposition rates and patterns as well as interactions with lung cells. If the ultrafine/Nanoparticles disaggregate upon interaction with alveolar lung fluids at sites of particle deposition (i.e., alveolar duct bifurcations), then they could behave as discrete individual Nanoparticles and may stimulate enhanced inflammatory cell recruitment and/or the particles could preferentially translocate to more vulnerable anatomical compartments of the lung. Alternatively, aggregated nanoparticle-types could behave as fine-sized particles. On the other hand most inhalation and instillation studies have been done with aggregated particles and in these instances, the NP (based on primary particle diameter) cause more inflammation.

In summary, there exists a paucity of relevant data on the pulmonary effects of inhaled Nanoparticles (Table 5). Particle size is an important factor but is only one of many variables that determine health effects. As a consequence, no general conclusions regarding nanoparticle toxicity can be made at the present time. Therefore, it is important that evaluations of safety and health risks of newly developed engineered nanoparticulates should be made following relevant testing on a case-by-case basis for each of the nanoparticle-types.

#### 4.3.2 The reticulo- endothelial system

The reticulo-endothelial system consists of cells that have the ability to phagocytose cellular debris, aged cells, pathogens and foreign substances including inert particles from the blood stream. Such cells include macrophages, monocytes, and specialised endothelial cells that line organs such as the liver, spleen and bone marrow. The reticulo-endothelial system located in the liver is exposed to all Nanoparticles absorbed from the gastrointestinal tract (GIT) into the cardiovascular system, since all blood exiting the GIT does so in the hepatic portal vein that directly perfuses the liver. The main function of this system is thought to be the removal and neutralization of any potential pathogens that enter the body from the GIT microflora. However, these cells have the capacity to phagocytose Nanoparticles. The consequences of nanoparticle uptake by these macrophages is not yet known, however there is evidence from in vitro studies that low solubility, low toxicity Nanoparticles such as carbon black and polystyrene stimulate the macrophages via reactive oxygen species and calcium signalling, to make pro-inflammatory cytokines such as tumour necrosis factor alpha [124]. Oxidative stress is known to inhibit hepatocyte function and bile formation [125], while pro-inflammatory cytokines are also associated with the pathology of liver disease. Hence, the impact of Nanoparticles on the liver and reticulo-endothelial system needs to be investigated.

#### 4.3.3 Cardiovascular effects and haematocompatibility

Ligand coated engineered Nanoparticles are being explored for decades as agents for molecular imaging or drug delivery tools. This has led to a considerable understanding of particle properties that can affect penetration in tissue without affecting tissue function. A size-dependent NP penetration in aorta vessel wall was noted after local delivery of polystyrene NP [126]. A more sophisticated approach for imaging of angiogenesis is achieved by integrin-targeted paramagnetic ironoxide NP [127]. Similarly cationic NP, including gold and polystyrene have been show to cause haemolysis and blood clotting, while usually anionic particles are quite non-toxic. This conceptual understanding maybe used to prevent potential effects of unintended NP exposure. Similarly, drug loaded Nanoparticles have been used to prolong half-life or reduce side-effects and have shown which particle properties need to be modified to allow delivery, while being biocompatible (review:[128] Also this know-how can help to develop engineered Nanoparticles for other applications that are with low hazard.

On the other hand, there is need to find explanations for the increased risk of patients with CV diseases upon exposure to PM and/or traffic. Cardiovascular effects of (inhaled) NP have been described only in human panel and animal studies, but the well-established cardiovascular effects of PM_10_, described in human epidemiological studies, has not yet been linked to the NP component. In addition, experimental animal studies with combustion NP do show that high exposures to diesel soot NP or other surrogate NP causes observable cardiovascular effects. However these are invariably seen in experimental animals given high doses, often by instillation into the lungs or the blood. Several toxicological studies have demonstrated that combustion and model NPs can gain access to the blood following inhalation or instillation and can enhance experimental thrombosis. Diesel particles instilled into hamster lungs also enhance thrombosis but it is not clear whether this was an effect of pulmonary inflammation or particles translocated to the blood [129,129-131]. High exposures to DEP by inhalation caused altered heart rate in hypertensive rats [132] interpreted as a direct effect of DEP on the pacemaker activity of the heart. Inflammation in distal sites has long been associated with destabilization of atheromatous plaques and instilled high doses of PM in Watanabe rabbits cause morphological evidence of atheromatous plaque destabilisation [133]. Ultrafine carbon black instilled into the blood has been reported to induce platelet accumulation in the hepatic microvasculature of healthy mice in association with prothrombotic changes on the endothelial surface of the hepatic microvessels [134] Inhalation exposure to diesel nanoparticles caused endothelial dysfunction measuerbal in the forearm as well as a decrease in stimulated tPA release

#### 4.3.4 CNS effects

As discussed previously inhaled Nanoparticles can gain access to the brain by two different mechanisms:

• transsynaptic transport after inhalation through the olfactory epithelium, and

• uptake through the blood-brain barrier

The first pathway has been studied primarily with model particles such as carbon, Au and MnO_2 _in experimental inhalation models. The second pathway has been the result of extensive research and particle surface manipulation in drug delivery, as an approach to try and get drugs to the brain [83,135] The latter studies suggest that the physiological barrier may limit the distribution of some proteins and viral particles after transvascular delivery to the brain, suggesting that the healthy BBB contains defence mechanisms protecting it from blood borne nanoparticle exposure. A number of pathologies, including hypertension and allergic encephalomyelitis, however have been associated with increased permeability of the BBB to Nanoparticles in experimental set ups. Conversely, the nanoparticle surface charges has been shown to alter blood-brain integrity [136] and need consideration as to their role in brain toxicity and brain distribution.

The use of paramagnetic Nanoparticles for MRI imaging of different cell types within neural tissue has proved useful experimentally[137], and it has been suggested that this might be useful in humans to track, for example, the development of stem cell grafts used to treat neurodegenerative diseases. However, the potential impact of Nanoparticles on human neuronal tissue is as yet not investigated in detail. Nanoparticles have been shown to induce the production of reactive oxygen species and oxidative stress and oxidative stress has been implicated in the pathogenesis of neurodegenerative diseases such as Parkinson's and Alzheimer's [138]. It is conceivable that the long term effects might include a decrease in cognitive function. Evidence for such effects is presented by studies in biopsies from city dwellers and Alzheimer's like pathology have demonstrated increased markers of inflammation and AB42-accumulation in frontal cortex and hippocampus in association with the presence of Nanoparticles [129]. Additionally, inhalation exposure of BALB/c mice to with particulate matter showed that activation of pro-inflammatory cytokines in the brain of exposed mice [139]. Whether this is due to the fraction of combustion Nanoparticles remains to be investigated.

#### 4.3.5 Dermal effects of NP

Particles with a size of approximately 50–500 nm are widely used in cosmetic products, in order to improve the homogeneity of the distribution of the formulations on the skin surface, or to act as UV filters against sun radiation. Some of the particles are included in the definition of NP as indicated above and are often used in sunscreens and skin care products for daily use. The concentration of the Nanoparticles in formulations is generally less than 3%. Sunscreens are applied onto the skin at a concentration of 1 mg/cm^2 ^or less. The particles act as "nanomirrors" on the skin and partly reflect the sunlight. The discussion on dermal effects of these NP mainly focussed on the question whether these particles are able to penetrate into or through the skin.

Because of their scattering properties, NP increases the optical pathway of UV photons entering the upper part of the horny layer. In this way, more photons are absorbed by the stratum corneum and by the applied organic filter substances. Therefore coated titanium dioxide Nanoparticles are commonly used as UV filter substances in commercial sunscreen products. In addition, modern sunscreens usually contain organic UV-filter substances such as butyl methoxydibenzoylmethane (BMDBM), 4-methylbenzylidene camphor (MBC). After topical application and equilibration, these UV-filters are located on the surface in the upper part of the stratum corneum where they form a protective layer [140,141] or especially in the case of titanium dioxide, they were reported not to enter the skin [91,93]. The efficacy of sunscreen products is characterized by the sun protection factor (SPF) [142]. Usually, the SPF of a formulation containing organic and inorganic filter compounds is higher than the sum of the sun protection factors of the individual UV-filter substances [143]. There is a synergistic (or additive) effect between organic and inorganic UV-filter substances.

### Effects of NP- summary

Both animal and human data suggest that NP are able to cause acute and chronic effects in the lung ranging from inflammation, exacerbations of asthma to genotoxicity and carcinogenesis. The tumour-related effects are unique to rats and have not been reported in other particle-exposed, rodent species such as mice or hamsters, under similar chronic conditions. Current epidemiological data in workers exposed to (pigmentary) TiO2 and CB do not show increased risks for lung cancer. Although particle size and surface area seems to be important particle parameters, currently the understanding of particle properties in relation to hazards is limited. In addition, it is important to delineate the pulmonary effects of ultrafine particles in rats at overload vs. non-overload conditions.

The emerging data on uptake of NP in the brain upon inhalation present a further challenge to toxicology and medicine to investigate the functional relevance of this translocation to CNS function and indirect systemic effects. The uptake of engineered NP through the blood-brain barrier is an intended effect in drug delivery and can only be achieved by very specific surface modifications.

The cardiovascular effects of NP may be related to both brain uptake as well as direct effects after various uptake pathways. Research in drug delivery has shown which engineered NP upon intravenous delivery have little effects on the cardiovasculature, and this know-how can be used to manufacture NP without these hazards in other applications of Nanotechnologies that may lead to exposure and uptake of NP.

NP are widely used in cosmetic preparations applied to human skin, such as sunscreens. Various NP preparations of zinc oxide or titanium oxide have been tested in vitro for percutaneous penetration, phototoxicity or photo-genotoxicity. At the present state of knowledge, there is little evidence that NP in cosmetic products may penetrate human skin and produce human systemic exposure. Overall, available data suggest that the human health risk from the dermal exposure to NP materials is low, but the published data set certainly needs extension.

## 5) Environmental impact of nanoparticles

Some Nanoparticles can occur naturally, through combustion or nucleation for instance, and this is the way they may impact on human health. It is estimated that 50,000 kg/year of nano-sized materials are being produced through these un-intended anthropogenic sources. In Chapter 4 the evidence for the role of Nanoparticles in the effects induced by ambient air pollution was reviewed. On the other hand, NP are increasingly manufactured and depending on the techniques used in manufacturing them, NP could be released in air, water, and ultimately contaminate soil and food products [144]. In 2003, Single-Walled and Multi-Walled Nanotubes had a worldwide production of 2954 kg. However, the Carbon Nanotechnology Research Institute (Japan) plans on expanding their production from ~ 1000 kg in 2003 to 120,000 kg per year within the next five years. Although current production of engineered nanomaterials is small, it is evident that production rates will accelerate exponentially in the next few years. Considering the tons of engineered nanomaterial planned for production, it is likely that some of these materials will enter the environment during the product's Life Cycle (manufacture, use, disposal). In addition to these specifically engineered nanomaterials, nano-sized particles are also being produced non-intentionally in diesel exhaust and other combustion processes.

Because of their extreme smallness, strong mobility and reactivity (Chapter 2), it appears relevant to evaluate risks related to NP transfer and persistence in the environment. Yet very little research has been done on environmental toxicology of engineered nanomaterials. This brief review serves as an overview of potential problems including **bioaccumulation**, **bio-toxicity **and **biodegradability **as well as potential benefits of nanotechnology in Environmental settings.

### 5.1 Impacts on environmental species

Few data are available for impacts of engineered nanomaterials on environmentally relevant species. No studies to date have been done on protists, fungi, plants, birds, reptiles, or amphibians, and the only mammalian studies have been carried out using laboratory species. Only one study has been done on fish and Arthropods, and no studies have been done on any other invertebrate phyla. Considering that invertebrates constitute 95–97% of all known animal species, there is a considerable lack of information on ecological endpoints. Only limited information is available for bacteria.

Data on environmental impacts of engineered nanomaterials are only available for un-coated, water-soluble colloids of fullerenes (nC_60_). These studies show that the *Daphnia magna *48 hour LC_50 _is approximately 800 ppb [145], making these fullerenes only moderately toxic. Largemouth bass (*Micropterus salmoides*) exposed to up to 1 ppm nC_60 _showed no signs of mortality or morbidity after 48 hours, although glutathione, a component of the anti-oxidant defence system, was depleted in the gill, and significant lipid peroxidation (LPO) was found in the brain [146]. Both the liver and gills had decreased LPO, possibly due to upregulation of repair enzymes [146]. Follow-up studies on these fish exposures using suppressive subtractive hybridization confirms the upregulation of repair enzymes as well as inflammatory response genes (data not yet published).

Filter-feeding organisms represent a unique target group for nano-particle toxicology. In the aquatic ecosystem, zooplankton and filter feeding invertebrates make up the basis of food webs. Zooplankton, such as rotifers and branchiopods, are so small (micron range) that they feed on nano-sized materials, including bacteria, viruses and organic macromolecules. At this nano-scale, water is a viscous liquid and many filtering apparatuses strain nanosized particles based not only on size, but also on surface chemistry [147]. Therefore changing nanomaterial surface chemistry to make them more biocompatible could ultimately lead to selective filtering and uptake by zooplankton and other selective filter feeding invertebrates such as the mole crab studied by Conova [147]. Any nano-sized material can be selectively consumed by zooplankton due to their size, or can be taken in by generalist filter feeders, such as the bivalves. Special considerations in terms of safety assessment should be made for the ability of zooplankton and other filter-feeding invertebrates to consume nano-sized materials. Many benthic invertebrates specialize in ingesting sediment and extracting organic material. The chemistry of most nano-materials predicts that engineered nanomaterials will tend to adsorb to sediments, creating another unique target group in the Benthos.

In addition to filter feeders, nano-sized materials may also influence Protozoan species. Recent studies have shown that in cell culture, fibroblasts can alter their direction of movement depending on whether nano-sized islands are in the way [148]. Fibroblasts produced more filopodia to sense and gather information, and were actively interacting with the 10 nm × ~ 150 nm wide islands on the cell culture plates [148]. The filopodia seem to be actively involved in sensing both the physical and chemical nature of the substrate, and filopodia direct the formation of lamellipodia which ultimately leads the cell to the island [148]. This type of interaction has been found in cell types of various species and different nano-sized substrates [148]. This implies that Rhizopoda, or other protist species, which use pseudopodia for locomotion, may be able to specifically interact with nanomaterials in their environment.

An additional potential target group are the **Photosynthetic Primary Producers**. Some impressive technologies have been developed for more efficient solar cells based on chlorophyll [149]. This technology is based on electron transfer from a synthetic chlorophyll analogue to a C_60 _in a carbon paste. It would be prudent to investigate whether fullerenes could also act as electron acceptors from natural chlorophyll, and whether fullerene in the environment could potentially uncouple photosynthesis. This is purely speculative, and the use of fullerenes to make more efficient solar cells would be extremely useful in breaking the dependence on non-renewable fossil fuels.

### 5.2 Bactericidal properties of nanomaterials

Several studies have reported bactericidal properties of fullerenes or modified fullerenes. Both gram negative and gram positive bacteria are highly sensitive to nC_60_, with a 48 hour LC_50 _in the 20 ppb range [150]. Dr. Pedro Alvarez (Rice University) is following up on this observation and is currently investigating the effects of nC_60 _on bacterial communities. In the study of largemouth bass described above, it was noticed that exposure water was visibly more clear than control water, indicating that beneficial bacteria were most likely eliminated from the water [146]. Modified fullerenes have also been specifically engineered to be anti-microbial [26], which would be beneficial in Medicine, but could potentially be detrimental if released into the environment, similarly to what is currently occurring with overuse of traditional antibiotics.

Not all nanomaterials are bactericidal. For example, un-coated and peptide-wrapped (as prepared by methods of Dieckmann et al. 2003)[151] and ssDNA-wrapped Single-Walled Carbon Nanotubes (SWNT) [152] are not toxic to E. coli up to ppm levels, which are the limits of their water solubility (Dr. Rockford Draper, University of Texas, personal communication). Therefore a broad generalization that all nano-materials are bactericidal is not appropriate. Each material and coating must be assessed on an individual basis for their potential to disrupt microbial ecology, and also for their potential environmental toxicity.

### 5.3 Stability of engineered nanomaterials and weathering of surface coatings

Fullerenes, nC_60_, are stable in aqueous medium up to 10 mM NaCl and 10 mM NaN_3 _solution [153], and in up to 3% NaCl solution and in Houston-area low-salinity bayou water which contains tannins (personal communication, Mason Tomson, Rice University). We have also found that nC_60 _is stable in Reconstituted Hard Water, consisting of 192 mg/L NaHCO_3_; 120 mg/L CaSO_4_-2H_2_O; 120 mg/L MgSO_4_; 8 mg/L KCl, pH 8.5 [146]. Other nanomaterials have wide solubilities in water, biological fluids, and other solvents [152,154-159], and would be expected to be soluble in environmentally relevant conditions and inside organisms. Of particular interest are nanomaterials that are to be used for personal care products such as sunscreens [160]. Other active ingredients in sunscreens and personal care products have been found at measurable levels in lakes in Europe and elsewhere, at levels where impacts on wildlife is to be expected [161]. If engineered nanomaterials are introduced in widely used personal care products, they will certainly enter the environment as well.

Nano-sized particles do not move far in environmental conditions. Studies have shown that size correlates with movement, where smaller nano-sized particles are easily adsorbed onto surfaces of sand grains and are therefore immobilized [162]. Biological transport could still occur from ingested sediments, but physical movement of nano-sized materials is restricted by its small size and propensity to adsorb onto surfaces.

### 5.4. Impact of surface modifications

Two ways to modify nanomaterials to make them more biocompatible and less toxic are by the use of various coatings and by covalent surface modifications. Although this is a valuable exercise in the laboratory, some studies have shown that these coatings and covalent surface modifications can be altered under UV exposure or exposure to oxygen in the air, and cause rapid cytotoxicity [163,164]. Therefore, although coatings and surface modifications may be critically important in drug-delivery devices, the likelihood of weathering under environmental conditions makes it important to study toxicity under UV and air exposure conditions. This is similar to the problem of studying polycyclic aromatic hydrocarbons (PAHs) in the laboratory, where UV-exposure activates PAHs and makes them more toxic (for examples, see [165,166])

### 5.5 Use of nanomaterials in bioremediation

This potential problem of UV activation can also be turned into a potential benefit. For example, one can use the photoactivity of nanomaterials for bioremediation. Studies with UV-irradiated TiO_2 _showed promise in removing organics from phenol solutions [167]. Others have also used nanomaterials for bioremediation. This included the removal of various organics (phenol, *p*-nitrophenol, salicylic acid) using nano-TiO_2 _[168]; the decomposition of a carbothioate herbicide (Molinate) by zero-valent iron [169]; the removal of hormones (E2, T, E1, P) via nano-filters due to adsorption to the large nano-filter surface area [170]; the removal of PAHs by amphiphilic polyurethane (APU) Nanoparticles [171]; and the removal of phenanthrene from aquifer material by amphiphilic polymer particles [172]. Bioremediation has also been field tested, where nanoscale redox active bimetallic (Fe/Pd) particles were able to reduce trichloroethylene contamination up to 96% in field trials. There are likely other bioremediation uses of nanomaterials, and this is not an exhaustive list.

Although nanomaterials may be used for bioremediation, a cautionary note should be made about injecting lipophilic, redox active compounds into the environment. The impact on non-charismatic meiofauna (as opposed to charismatic megafauna at which most of our environmental legislation is aimed) needs to be considered. In addition, as noted by Lecoanet and Wiesner (2004) [162], nanosized materials may not migrate through soils at rapid enough rates to be valuable in bioremediation. Future laboratory and field trials will help clear up the line between bioremediation and biocontamination.

## Summary

Production of engineered nanomaterials on the order of thousands of tons by 2007 makes it very likely that these materials will enter the environment through production, manufacture, use, or disposal of products. There is an almost complete lack of data on bioaccumulation, bio-toxicity and biodegradation of NP in environmentally relevant species. There is also limited study of the weathering potential of both coatings and covalent surface modifications. Early studies indicate the fullerenes and their derivatives may be toxic in some species (fish, daphnia and bacteria), while other nanomaterials (SWNT) have limited toxicity to bacteria. Therefore no blanket statements about toxicity of nano-sized materials can be made at this time. Potential benefits of nanotechnology in the environment include uses in bioremediation and increasing efficiency of fuel cells and solar cells to decrease our dependence on fossil fuels (which have known toxic impacts on the environment).

**Table 2:** Global R&D expenditure [$M]

**Country/Region**	**1997**	**2002**
USA	432	604
Western Europe	126	350–400
Japan	120	750
South Korea	0	100pa (for 10 yrs)
Taiwan	0	70
Australia	0	40
China	0	40
Rest of world	0	270

see

**Table 3:** Public funds for R&D in nanoscience and nanotechnologies

**Country**	**Public funding**
Europe	Current funding ~ €1 B, largely from national and regional programmes
Japan	$400 M (2001); $800 M (2003); $1 B (estimated for 2004)
USA	$750 M (2003); $3.7 B (2005–2008, excluding defence)

Data source:

**Table 4:** Surfactants commonly used to stabilise Nanoparticles during their synthesis.

**Substance**	**Dispersible in:**
4-Dimethylaminopyridine	Water
Mercaptoundecaneacid	Water
Thiols (e.g. Dodecanethiol)	Unpolar solvents like Hexane, Toluene, Chloroform, Acetone
Tetraalkylhalogenides	Unpolar solvents like Hexane, Toluene, Chloroform, Acetone
Fluoralkanes	Unpolar solvents like Hexane, Toluene, Chloroform, Acetone
Trialkoxy-Silanes and derivates thereof	Water, Alcohol
Phosphorous containing substances like Ph_2_PC_6_H_4_SO_3_Na	pH dependent in water or unpolar solvents
Aminoalkanes and derivates thereof	Dependent on functionalisation unpolar solvents or Alcohol

These substances are likely to be on the surface throughout the lifetime of the particle

**Table 5:** Comparison of attributes of lung overload in rats vs. larger mammals such as dogs and primates (nonhuman and human) for particles with low solubility

Classical attributes and sequelae of lung overload in rats	Rats	Dog, monkey and man
Chronic pulmonary inflammation	yes	not certain
Hyperplasia of macrophages and epithelial cells	yes	not certain
Altered pulmonary clearance (overwhelmed) macrophage mediated clearance	yes	probably not
Large alveolar burdens of particles	yes	probably not
Increased interstitialization of deposited particles	yes	yes- greater % than rat
Increased translocation of particles from lung to thoracic lymph nodes	probably	probably
Interstitial lung disease (fibrosis)	yes	yes but less severe
Production of lung tumours	yes	no
